# Does value-based prioritization at working memory enhance long-term memory?

**DOI:** 10.3758/s13421-024-01532-9

**Published:** 2024-02-20

**Authors:** A. L. Atkinson, A. H. Waterman, R. J. Allen

**Affiliations:** 1 Department of Psychology, Lancaster University, Bailrigg, Lancaster UK; 2 School of Psychology, University of Leeds, Leeds, UK

**Keywords:** Working memory, Long-term memory, Attention, Reward, Prioritization

## Abstract

Research has demonstrated that individuals can direct their attention to valuable information in both working memory and long-term memory tasks with observable effects on performance. However, it is currently unclear whether prioritising an item for a working memory task automatically translates into a boost at long-term memory. This was examined in two experiments using relatively short (250 ms per item; Experiment 1) and longer (500 ms per item; Experiment 2) encoding times. Participants first completed a visual working memory task, in which they were presented with series of photographs of everyday objects. Following a brief delay (1,000 ms), they completed a four-alternative forced-choice test. Prior to encoding, participants were informed of the point values associated with each item. In some trials, the first item in the sequence was worth more points than the rest. In other trials, all items were equally valuable. After a filled delay, participants completed a surprise long-term memory task. At working memory, a value effect was reliably observed on recognition accuracy, along with some evidence of faster response times for high-value items. However, there was little consistent evidence of this effect automatically persisting into long-term memory. Thus, the benefits of attentional prioritization in working memory do not always translate into longer-term performance. More broadly, this provides further evidence that manipulations that enhance working memory performance do not necessarily enhance long-term memory.

**Supplementary Information:**

The online version contains supplementary material available at 10.3758/s13421-024-01532-9.

## Introduction

Working memory (WM) refers to a limited-capacity system that enables information to be stored in a temporary state in heightened accessibility for use in ongoing information processing (e.g., over a timeframe of seconds; Cowan, 2017). In contrast, long-term memory (LTM) is much larger in capacity (Brady et al., 2008; Standing, 1973) and operates over considerably longer timeframes. Although it is generally agreed that WM and LTM are related and interactive, their precise relationship remains under debate (e.g., Cowan, 2019; Forsberg et al., 2021). One important question is whether WM processes impact upon the formation of longer-term memories (e.g., Forsberg et al., 2021, 2022; Hartshorne & Makovski, 2019; Loaiza & Souza, 2021; Loaiza et al., 2023). Indeed, it has been demonstrated that several WM processes affect LTM, including attentional refreshing (Camos & Portrat, 2015) and consolidation (Cotton & Ricker, 2021). However, not all processes or manipulations that enhance WM consistently improve LTM (Camos & Portrat, 2015; Overkott & Souza, 2022). For instance, although verbal rehearsal is widely assumed to enhance WM (but see Souza & Oberauer, 2018, 2020), there is little evidence that it enhances LTM (Camos & Portrat, 2015). As such, it is important to identify processes or manipulations at WM that do, and do not, affect performance at LTM.

One question that has received considerable interest in both the WM and LTM literatures in recent years has been the extent to which individuals can prioritize particularly valuable information (e.g., indicated using notional points). In some studies, high-value items have been compared to low-value items within the same condition (i.e., a condition in which different items are more or less valuable; Allen & Ueno, 2018; Hu et al., 2014, 2016; Hitch et al., 2018, 2020). In other studies, high-value items have been compared to a separate condition in which all items are equally valuable (Allen et al., 2021; Atkinson et al., 2018, 2019, 2021, 2022; Hu et al., 2023; Sandry & Ricker, 2020; Sandry et al., 2014). Value effects are robust in working memory when participants are informed of the values in advance of encoding, with effects demonstrated using a variety of materials (e.g., visual material (shapes), visually presented verbal information (words), auditorily presented verbal information (digits), tactile information, and cross-modal binding of odour-visual information; e.g., Atkinson et al., 2021; Hu et al., 2016; Johnson & Allen, 2023; Roe et al., 2024; Sandry & Ricker, 2020; Sandry et al., 2014), retrieval methods (e.g., cued recall, recognition, colour reproduction; Atkinson et al., 2022; Hu et al., 2016; Hu et al., 2023; Sandry & Ricker, 2020; Sandry et al., 2014), and age groups (e.g., Allen et al., 2021; Atkinson et al., 2019; Hu et al., 2016). Separately, a value effect has also been consistently observed in LTM, with items that are assigned a higher value remembered better than lower-value items (Adcock et al., 2006; Castel et al., 2002, 2011; Gruber & Otten, 2010; Murty & Adcock, 2014; Shigemune et al., 2010; Spaniol et al., 2014; Wittmann et al., 2005; Yin et al., 2021; see Knowlton & Castel, 2022).

Whilst these streams of research have remained relatively distinct, recent studies have begun to explore whether prioritizing an item for a WM test influences LTM (Jeanneret et al, 2023; Sandry et al., 2020). These studies have produced somewhat mixed results. For example, Jeanneret et al. (2023) provided value information immediately following item presentation (i.e., retrospectively), using simultaneous displays of four to-be-remembered items on each visual WM trial. In this study, the high-value item was compared to lower-value items in the same condition. They found that value affected WM performance but had no impact on a surprise LTM test. Sandry et al. (2020) conducted an experiment with a similar aim, albeit using a somewhat different methodology, comparing memory for a high-value item to a different condition in which all items were equally valuable. They found that items associated with a higher value as part of a sequentially presented verbal WM test were remembered better on a surprise LTM test. However, this was only observed for items that were not tested during the preceding WM phase, with no boost observed for items that had been tested at WM. These mixed findings contrast with evidence that visual cue-based attentional manipulations (that predict which item will be assessed at retrieval) enhance LTM performance (e.g., Jeanneret et al., 2023; Reaves et al., 2016; Strunk et al., 2018).

Given these mixed findings concerning the effects of value-based prioritization at WM on LTM performance, further research is needed to establish the reliability of any such impacts. The current study addressed this in two experiments. In each experiment, participants were presented with sequences of four images of everyday objects in each WM trial. Participants were told either that the first item was more valuable than the rest (differential probe value) or that all the items were equally valuable (equal probe value). After a brief delay (1,000 ms), participants completed a four-alternative forced-choice (4-AFC) test in which they had to identify the item that had been presented during the encoding phase. Approximately 10 minutes after the end of the WM task, participants completed a surprise LTM test. Half of the items tested during this phase had been assessed at WM, whilst half had not been tested. This allowed us to explore whether the emergence of probe value effects at LTM differs depending on whether the item was tested at WM. Response times (RTs) were also measured at both the WM and the LTM phases. Experiment 1 used a short presentation time (250 ms per item; Hu et al., 2014, 2016; Hitch et al., 2018), whilst Experiment 2 examined the effects using a longer presentation time (500 ms per item; Allen et al., 2021; Atkinson et al., 2018, 2019; Sandry et al., 2014, 2020; Sandry & Ricker, 2020).

Based on previous research, we expected participants to respond more accurately and faster in the WM test for the high-value item relative to performance at the same serial position (SP) in a condition in which all items were equally valuable. This should emerge alongside no overall main effect of priority condition, as improved performance on the high-value item is typically accompanied by performance decrements on the low-value items in this condition (e.g., Atkinson et al., 2018; Hu et al., 2014; Hitch et al., 2020). Given the mixed existing findings (Jeanneret et al., 2023; Sandry et al., 2020), we did not have any strong predictions concerning the effect of value at LTM. However, if selective prioritization in WM automatically leads to a longer-lasting representation, this may be detectable in an advantage for the high-value item (relative to an equal-value condition) on the surprise delayed-memory test.

## Experiment 1

### Method

#### Participants

Power analysis was conducted using G*Power (Faul et al., 2007). In Atkinson et al. (2018; Experiment 1), the effect size for the probe value effect (Cohen’s *d*) was 1.33. Based on the assumption that any effect at LTM is likely to be smaller in size, we halved this effect size when calculating the number of participants needed for the current study. Based on an effect size of *d* = .665 and alpha = 0.05, it was estimated that 21 participants would provide 90% power. Thirty-six participants completed the experiment (*M. age* = 19.74 years, *SD* = 1.68 years; range = 18–26 years; eight males). Participants were fluent English speakers, had normal or corrected-to-normal vision, and had no known learning difficulties. They were reimbursed with course credit or cash. The study was approved by the School of Psychology Ethics Committee at the University of Leeds (Ethics reference number PSYC-626).

#### Design, materials and procedure

**Fig. 1:**
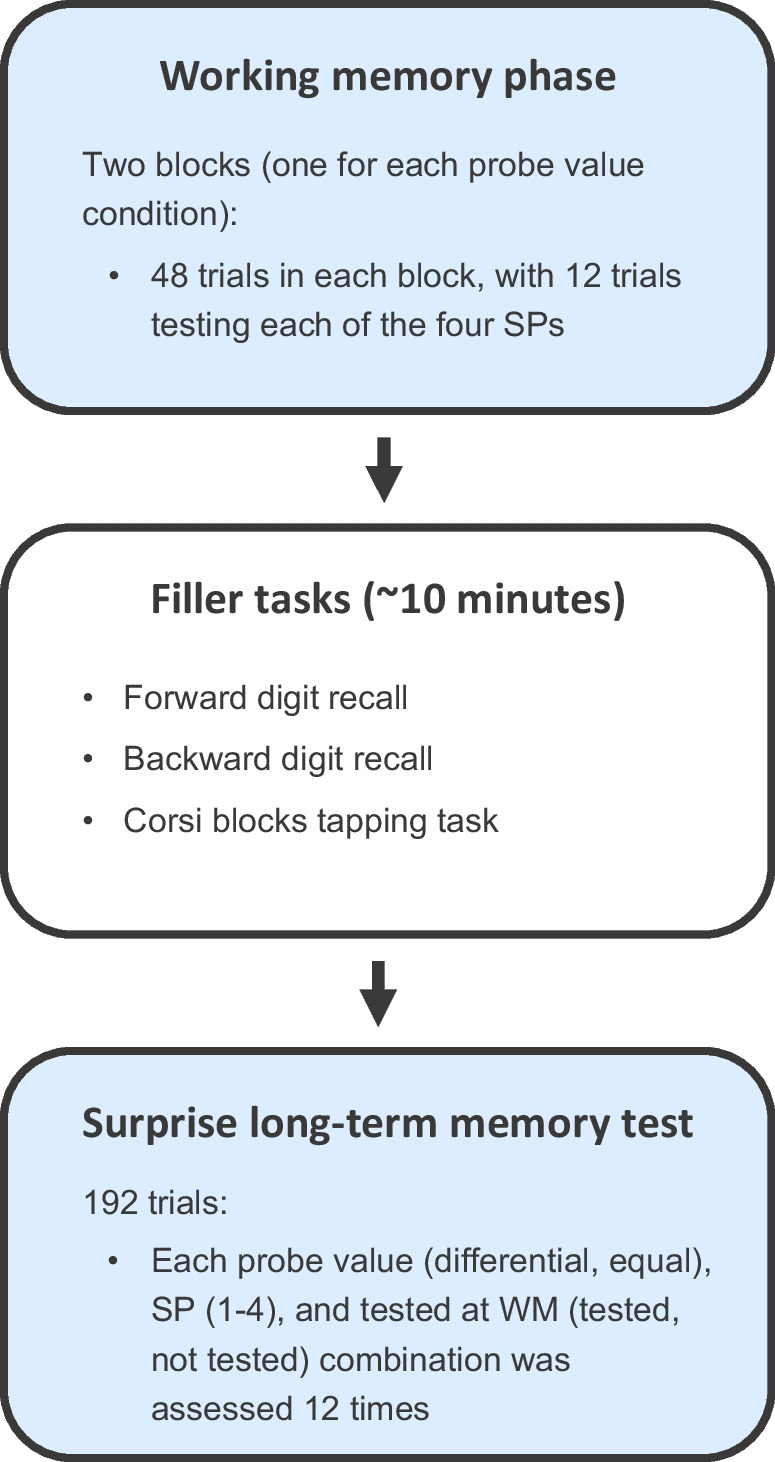
The structure of the experiment in Experiments 1 and 2. *SP* serial position, *WM* working memory

The study comprised two main parts: a WM phase and a LTM phase. At the WM phase, a 2 (Probe value: differential vs. equal) × 4 (SP: 1–4) within-subject design was employed. At LTM, a 2 (Probe value: differential vs. equal) × 4 (SP: 1–4) × 2 (Tested-at-WM: tested vs. not tested) within-subjects design was employed. At both the WM and LTM test phases, the dependent variables were accuracy (proportion correct) and RT. The experiment was completed as part of one in-person session, taking approximately 60 minutes. Participants were tested individually. The structure of the session is displayed in Fig. 1.

Participants first completed a WM phase. This comprised two blocks (one for each of the probe value conditions), each containing 48 trials. The order of blocks was fully counterbalanced across participants. Within the blocks, each of the four SPs was assessed 12 times. The order of the SP trials within each block was randomised such that participants could not predict which item would be tested.

**Fig. 2:**
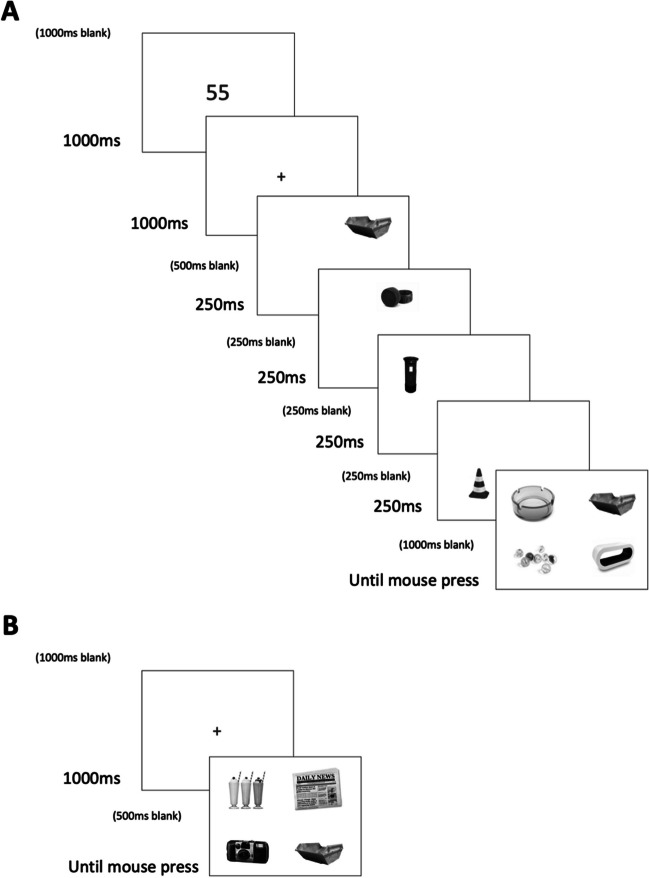
The schematic used in the working memory (**A**) and long-term memory (**B**) trials in Experiment 1. Figure not to scale

The paradigm used in the WM phase is displayed in Fig. 2A. Participants were first presented with a blank screen for 1,000 ms, followed by a randomly generated number between 20 and 99 for 1,000 ms. Participants repeated this number aloud until the retrieval phase to disrupt verbal rehearsal (Baddeley, 1986). A fixation cross was then displayed for 1,000 ms, followed by a blank screen for 500 ms. Next, participants were presented with four images of everyday objects for 250 ms, separated by an inter-stimulus interval (ISI) of 250 ms. Images were taken from two large datasets: the Bank of Standardized Stimuli (BOSS; version 2; Brodeur et al., 2010, 2014) and Brady et al. (2008). Where the same object appeared in both databases, one of these was removed to ensure that each object presented was distinct. The images were presented in greyscale as pilot work revealed at-ceiling performance when coloured images were used. The images presented were selected pseudo-randomly for each participant, with the constraint that each image could only be presented once during the entire experiment. Each image appeared at one of eight equally spaced locations positioned around an imaginary circle of radius 5.66º, located at the centre of the screen. The positions used in each trial were selected pseudo-randomly, with the constraint that no position could be used more than once within a trial. The images measured approximately 4º, based on a viewing distance of 50 cm. There was a retention interval of 1,000 ms following item presentation. Participants were then presented with one item from the encoding phase and three lures (each measuring 4º) that were not used elsewhere in the experiment. Items were presented at corners of an imaginary 8º wide square, located at the centre of the screen. Participants had to click on the item that had been presented during the encoding phase using a computer mouse. The images remained on-screen until the participant responded. Participants were told that accuracy was more important, but that they should respond as quickly as possible.

Probe value instructions were provided before the encoding phase. In the differential probe value condition, participants were informed that they would receive 4 points if they were asked about the first item and they responded correctly. If they were asked about any other item and they responded correctly, they would get 1 point. In the equal probe value condition, all the items were worth the same number of points (1 point). Points were notional and were not associated with any physical reward (e.g., money). At the start of each probe value block, participants completed two practice trials to familiarise themselves with the task. Reminders of the probe value manipulation were presented after every 12 trials.

Following the WM phase, participants completed three filler tasks: forward digit recall (FDR), backward digit recall (BDR), and the Corsi blocks tapping task, with the order counterbalanced across participants. WM tasks were used as a filler to reduce the likelihood of participants guessing that a LTM test would follow. This took approximately 10 minutes in total. Further details about these tasks are presented in the Online Supplementary Materials (OSM).

After the filler tasks, participants completed a surprise LTM test where memory for the items presented during the WM task was assessed (see Fig. 2B). Within each trial, a blank screen was presented for 1,000 ms, followed by a fixation cross for 1,000 ms, and then a blank screen for a further 500 ms. After this, four images of everyday objects were presented at corners of an imaginary 8º wide square located at the centre of the screen. One of the images had been presented during the encoding phase of the WM task and three were new lures that had not been presented at any other point during the experiment. Participants had to select which item had been presented during the encoding phase using a computer mouse. In the ‘tested’ trials, the target item had also appeared as the target during the WM test phase. In the ‘not tested’ trials, the item tested had not appeared as the target during the WM test phase. As with the WM phase, participants were told that accuracy was more important, but that they should try to respond as quickly as possible. Each probe value (differential, equal), SP (1–4), and tested-at-WM (tested, not tested) combination was assessed 12 times. This task therefore comprised 192 trials in total, with the order of trials randomised. Participants were asked to take a short break after every 40 trials.

After the LTM phase, participants completed a short questionnaire (see OSM). This asked them whether they predicted the LTM task (yes/no). They were also asked the extent to which they thought about the images between the WM and LTM tests. This was measured on a 7-point Likert scale, where 1 reflected ‘not at all’ and 7 reflected ‘all of the time’. Participants were also asked whether they believed prioritization helped or harmed their memory for the more valuable item and the less valuable items in both the WM and LTM phases, using a 9-point Likert scale (where 1 = large negative effect, 5 = no perceived effect, and 9 = large positive effect). Responses were recoded by subtracting five from each value, such that -4 reflects a large negative effect, 0 indicates no effect, and 4 reflects a large positive effect. Responses regarding whether participants predicted the LTM test and the extent to which they thought of the items during the interval are reported briefly in the *Data analysis* sections, whilst opinions concerning the effects of prioritization are presented in the OSM.

#### Data analysis

Data for both experiments is available on the Open Science Framework at https://osf.io/6w824/.

As the experiment primarily aimed to assess how prioritizing an item for a WM test would affect performance in a surprise LTM task, participants who anticipated the second memory task were excluded from all analyses (Murayama & Kuhbandner, 2011). This resulted in two out of 36 participants (6%) being excluded. The analysis was therefore conducted on data for 34 participants. Generally, these participants did not report thinking about the objects much in the interval between the WM and LTM tests (*M* = 2.34, *SE* = 0.27, where 1 = not at all and 7 = all of the time).

Across all analyses, proportion correct was used as the primary outcome measure, with RT computed as a secondary outcome measure. Accuracy reflects the proportion of trials in which participants responded correctly. RT was measured (in ms) from the onset of the test stimuli until the participants responded using the mouse. RTs for incorrect responses were discarded. This resulted in the removal of 715/3,264 trials in the WM phase (21.91%) and 3,297/6,528 in the LTM phase (50.51%). RTs above 20,000 ms (20 s) were then removed, followed by RT trimming. RTs that fell 2.5 SDs above or below the mean for each condition for each participant were excluded. These steps resulted in the exclusion of 30/3,264 data points in the WM phase (0.92%) and 7/6,528 data points in the LTM phase (0.11%).

Across all analyses, both frequentist and Bayes factor (BF) analysis was conducted. BF analysis indicates the strength of evidence for the presence or absence of an effect. Bayesian ANOVAs were conducted using the ‘BayesFactor’ package (Morey & Rouder, 2022) in R (R Core Team, 2022). Default priors were used, and the number of iterations were set to 500,000. All models were computed, such that a model could contain an interaction in the absence of main effects. In addition to reporting the best model, we report BFs for individual main effects and interactions. These Bayes factors were computed by re-running the model with the which_model argument set to ‘top’. This compares a model that omits a main effect/interaction to the model containing all main effects and interactions. This produces *BF*_*01*_ values, which indicates evidence of no effect. *BF*_*10*_ values were derived by inverting the (*BF*_*01*_) values (1/ *BF*_*01*_)_*.*_ A *BF*_*10*_ value above 1 provides evidence for an effect, whereas a *BF*_*10*_ value below 1 provides evidence of no effect. For ease of interpretation, when *BF*_*10*_ is below 1, we also present *BF*_*01*_. Frequentist analysis was conducted in R, using the afex (Singmann et al., 2022) and emmeans (Lenth, 2022) package. Post hoc comparisons for the frequentist ANOVAs were corrected using Bonferroni-Holm. We primarily draw conclusions based on p-values, but we draw readers’ attention to any discrepancies that would result from interpreting p-values versus BFs.

### Results

#### Working memory

##### Accuracy

**Fig. 3:**
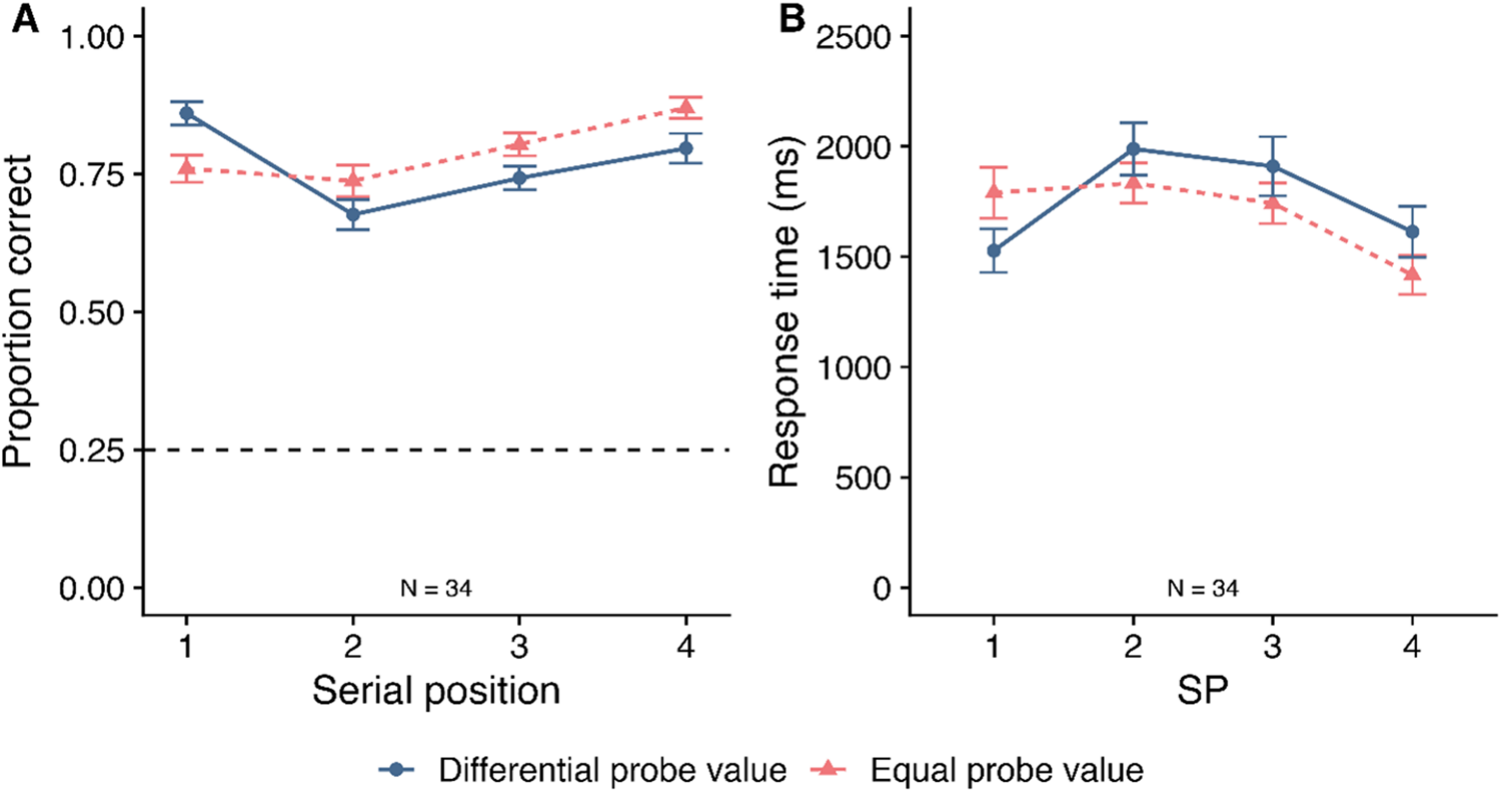
Mean proportion correct (**Panel A**) and mean response time (**Panel B**) at working memory in Experiment 1 as a function of probe value and serial position (SP). Error bars display SE. The dashed grey line at 0.25 in Panel A reflects chance rate

Mean proportion correct (and SE) is displayed in Fig. 3A as a function of probe value and SP. A 2 (Probe value: Differential vs. Equal) – 4 (SP: 1–4) within-subjects ANOVA was conducted. This revealed no significant effect of probe value (*F*(1, 33) = 3.25, *MSE* = 0.01, *p* = .081*,* ${\eta }_{p}^{2}$ = .09; *BF*_*10*_ = 0.42, *BF*_*01*_ = 2.40), but a significant effect of SP (*F*(3, 99) = 10.73, *MSE* = 0.02, *p* < .001*,* ${\eta }_{p}^{2}$ = .25; *BF*_*10*_ > 10,000). Pairwise comparisons (corrected using Bonferroni-Holm) revealed that performance at SP2 (*M* = .71, *SE* = .02) was significantly worse than performance at SP1 (*M* = .81, *SE* = .02; *p* = .001; *BF*_*10*_ = 230.39), SP3 (*M* = .77, *SE* = .02; *p* = .035; *BF*_*10*_ = 6.51), and SP4 (*M* = .83, *SE* = .02; *p* < .001; *BF*_*10*_ = 7028.16). Performance at SP3 was also significantly worse than performance at SP4 (*p* = .017; *BF*_*10*_ = 26.96). There was also a significant interaction between probe value and SP (*F*(3, 99) = 7.81, *MSE* = 0.02, *p* < .001*,* ${\eta }_{p}^{2}$ = .19; *BF*_*10*_ = 228.30). The BF analysis revealed the best model contained a main effect of SP and an interaction between probe value and SP (*BF*_*10*_ > 10,000 relative to the null model containing participant only).

To understand the interaction, Bonferroni-Holm-corrected post hoc tests were conducted to examine the effect of probe value at each SP. At SP1, there was significantly higher accuracy in the differential probe value condition (*M* = .86, *SE* = .02) than in the equal probe value condition (*M* = .76, *SE* = .02; *t*(33) = 3.37, *d* = 0.58, *p* = .008; *BF*_*10*_ = 17.99). In contrast, at SP4, accuracy was significantly higher in the equal probe value condition (*M* = .87, *SE* = .02) than in the differential probe value condition (*M* = .80, *SE* = .03; *t*(33) = -2.82, *p* = .024, *d* = -0.48, *BF*_*10*_ = 5.23). There were no significant differences at SP2 (*t*(33) = -1.94, *p* = .074, *d* = -0.33, *BF*_*10*_ = 0.98; *BF*_*01*_ = 1.02) or SP3 (*t*(33) = -2.17, *p* = .074, *d* = -0.37, *BF*_*10*_ = 1.45).

##### *Response times*

Mean RT (and SE) is displayed in Fig. 3B as a function of probe value and SP. A 2 (Probe value: differential vs. equal) × 4 (SP: 1–4) within-subjects ANOVA revealed no significant main effect of probe value (*F*(1, 33) = 1.18, *MSE* = 234983.63, *p* = .284*,* ${\eta }_{p}^{2}$ = .04; *BF*_*10*_ = 0.30; *BF*_*01*_ = 3.32). There was a significant main effect of SP (GG-corrected *F*(2.26, 74.68) = 10.45, *MSE* = 268620.40, *p* < .001*,* ${\eta }_{p}^{2}$ = .24; *BF*_*10*_ > 10,000). Bonferroni-Holm-corrected pairwise comparisons revealed that RT at SP2 (*M* = 1911, *SE* = 99.3) was significantly slower than at SP1 (*M* = 1659, *SE* = 93.0; *p* = .001; *BF*_*10*_ = 112.18) and SP4 (*M* = 1515, *SE* = 99.1; *p* = .001; *BF*_*10*_ > 10,000). Furthermore, RT at SP3 (*M* = 1826, *SE* = 102.9) was significantly slower than at SP4 (*p* = .001; *BF*_*10*_ = 2075.45). There was also a significant interaction between probe value and SP (*F*(3, 99) = 8.59, *MSE* = 94452.74, *p* < .001*,* ${\eta }_{p}^{2}$ = .21; *BF*_*10*_ = 14.71). The BF analysis showed that the best model included a main effect of SP and an interaction between probe value and SP (*BF*_*10*_ > 10,000 relative to the null model containing participant only).

Bonferroni-Holm-corrected post hoc tests were conducted to examine the effect of probe value at each SP. At SP1, there were no significant differences between probe value conditions after correction, although this difference did approach significance (Differential probe value *M* = 1527, *SE* = 98.7; Equal probe value *M* = 1790, *SE* = 115.2; *t*(33) = -2.45, *p* = .059, *d* = -0.42, *BF*_*10*_ = 2.45). There were also no significant differences at SP2 (*t*(33) = 2.04, *p* = .100, *d* = 0.35, *BF*_*10*_ = 1.14) or SP3 (*t*(33) = 1.63, *p* = .112, *d* = 0.28, *BF*_*10*_ = 0.61, *BF*_*01*_ = 1.64). At SP4, RT was significantly faster in the equal probe value condition (*M* = 1417, *SE* = 88.3) than in the differential probe value condition (*M* = 1613, *SE* = 114.9; *t*(33) = 3.76, *p* = .003, *d* = 0.65; *BF*_*10*_ = 46.57).

#### Long-term memory

##### Accuracy

**Fig. 4:**
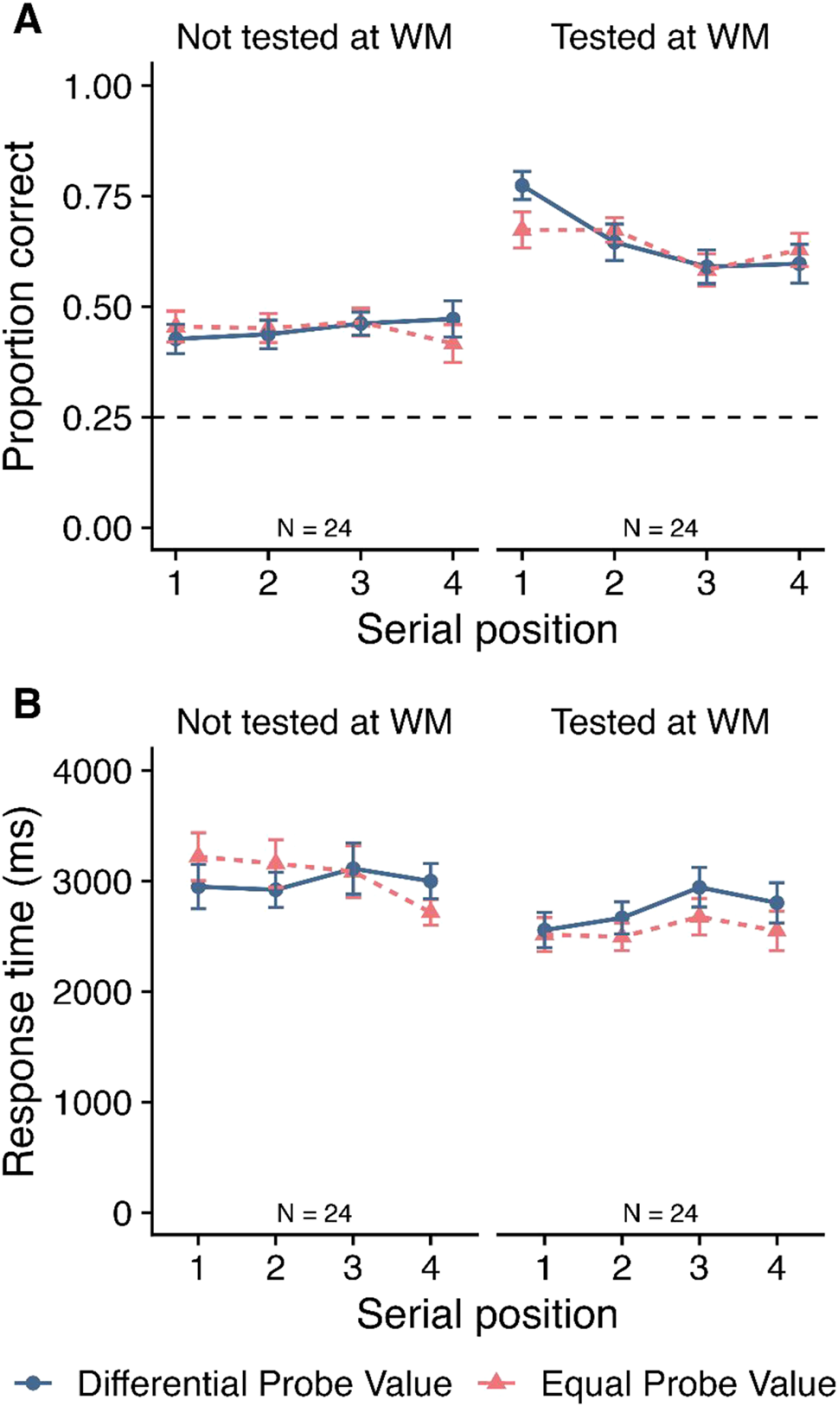
Mean proportion correct (**Panel A**) and mean response time (**Panel B**) at long-term memory in Experiment 1 as a function of probe value, serial position (SP), and tested-at-working memory (WM). Error bars show SE. The dashed grey line at 0.25 in Panel A reflects chance rate

Mean accuracy in the LTM phase is displayed in Fig. 4A as a function of probe value, SP and tested-at-WM. A 2 (Probe value: differential vs. equal) × 4 (SP: 1–4) × 2 (Tested-at-WM: not tested vs. tested) within-subjects ANOVA was conducted. This revealed no significant effects of probe value (*F*(1, 33) = 0.01, *MSE* = 0.02, *p* = .910*,* ${\eta }_{p}^{2}$ < .01; *BF*_*10*_ = 0.10; *BF*_*01*_ = 10.37) or SP (*F*(3, 99) = 0.28, *MSE* = 0.02, *p* = .837*,* ${\eta }_{p}^{2}$ = .01; *BF*_*10*_ = 0.01; *BF*_*01*_ = 115.50). There was a significant main effect of tested-at-WM (*F*(1, 33) = 208.90, *MSE* = 0.03, *p* < .001*,* ${\eta }_{p}^{2}$ = .86; *BF*_*10*_ > 10,000), with higher accuracy for items that had been tested at WM (*M* = 0.60, *SE* = 0.02) relative to items that had not being tested (*M* = 0.39 *SE* = 0.01). There were no two-way interactions between probe value and SP (*F*(3, 99) = 0.34, *MSE* = 0.02, *p* = .796*,* ${\eta }_{p}^{2}$ = .01; *BF*_*10*_ = 0.02; *BF*_*01*_ = 41.41) or probe value and tested-at-WM (*F*(1, 33) = 1.40, *MSE* = 0.02, *p* = .246*,* ${\eta }_{p}^{2}$ = .04; *BF*_*10*_ = 0.29; *BF*_*01*_ = 3.47), although a significant interaction did emerge between SP and tested-at-WM (*F*(3, 99) = 4.21, *MSE* = 0.02, *p* = .008, ${\eta }_{p}^{2}$ = .11; *BF*_*10*_ = 2.25); see OSM for post hoc tests). The three-way interaction between probe value, SP and tested-at-WM was not significant (*F*(3, 99) = 0.46, *MSE* = 0.02, *p* = .708*,* ${\eta }_{p}^{2}$ = .01), with BF analysis in favour of no effect (*BF*_*10*_ = 0.07; *BF*_*01*_ = 15.24). The BF analysis indicated that the best model included a main effect of tested-at-WM and an interaction between SP and tested-at-WM (*BF*_*10*_ > 10,000 relative to the null model containing participant only).

##### Response times

One participant was excluded from this analysis as they had an empty cell resulting from all data for that condition being excluded. This analysis was therefore conducted on data from 33 participants. RTs are displayed in Fig. 4B a function of probe value, SP and tested-at-WM. A 2 (Probe value: differential vs. equal) × 4 (SP: 1–4) × 2 (Tested-at-WM: yes vs. no) within-subjects ANOVA revealed no significant main effect of probe value (*F*(1, 32) < 0.01, *MSE* = 287446.30, *p =* .976, ${\eta }_{p}^{2}$ < .01; *BF*_*10*_ = 0.10; *BF*_*01*_ = 10.31) or SP (*F*(3, 96) = 2.18, *MSE* = 390197.96, *p* = .095, ${\eta }_{p}^{2}$ = .06; *BF*_*10*_ = 0.13; *BF*_*01*_ = 7.48). There was a main effect of tested-at-WM (*F*(1, 32) = 45.09, *MSE* = 462958.92, *p* < .001, ${\eta }_{p}^{2}$ = .59; *BF*_*10*_ > 10,000), with participants responding significantly faster when the item was tested at WM (*M* = 2691, *SE* = 129) relative to when it was not tested at WM (*M* = 3089, *SE* = 155). No significant interactions emerged (*F* ≤ 1.55, p ≥ .216, ${\eta }_{p}^{2}$ ≤ .05, *BF*_*10*_ ≤ 0.17; *BF*_*01*_* ≥* 5.84). The BF analysis revealed that the best model included tested-at-WM (*BF*_*10*_ > 10,000 relative to the null model containing participant only).

### Discussion

The current study investigated whether prioritizing a more valuable item for a WM task resulted in a durable boost that could be observed at LTM. A value effect was apparent in the WM task, with participants responding more accurately at SP1 when it was assigned with a higher value. This is in line with previous findings that have demonstrated an effect of probe value on WM performance (e.g., Atkinson et al., 2018, 2022; Hitch et al., 2018; Hu et al., 2014, 2023; Sandry et al., 2020). Here, we extend the effect to a recognition task using images of real-world objects. This boost was accompanied by significant costs at SP4. This supports a resource trade-off account, whereby directing attention to a particularly valuable item often comes at a cost to the less valuable items (e.g., Atkinson et al., 2018; Brissenden et al., 2023; Hu et al., 2014; Sandry et al., 2020). Performance for equal-value items was also numerically higher than differential value items at SP2 and SP3, but this difference did not reach significance after correction (*p* = .074 for both SPs). Observation of larger costs to the final item are in line with previous research (e.g., Atkinson et al., 2018). One possibility is that the cost to SP2 and SP3 may have been offset somewhat by the prioritization boost spreading to nearby items. Alternatively, it is possible that the costs at these positions are reliable but small, with the sample size used in this study not large enough to detect them. Indeed, this is possible given that the sample size calculations in the current study were based on the boost to SP1, rather than on costs to other SPs. We return to this issue later. There was also a trend towards faster responding for the high-value item, though the difference between differential and equal-value conditions at SP1 was not significant following correction (*p* = .059), with BF analysis providing only ambiguous evidence of an effect (*BF*_*10*_ = 2.45).

At LTM, there were no observable effects of value-driven prioritization. This was consistent regardless of whether the item had been tested during the WM phase or not. In contrast, a large testing effect was apparent, with items tested at WM being recalled more accurately than items that were not tested at WM. Given the target item was presented during the WM test phase alongside three lures, this effect is likely to reflect a combination of the item being tested and presented on-screen again during the retrieval phase. Nevertheless, this provides further evidence that processes at WM can influence LTM performance (Camos & Portrat, 2015; Cotton & Ricker, 2021).

## Experiment 2

Outcomes from Experiment 1 were clear in indicating accuracy benefits of value-based prioritization on WM, but not on a surprise LTM test. Experiment 2 was therefore designed with the aim of establishing whether these patterns of transient value effects would replicate, when providing more time during initial WM encoding. Experiment 1 presented each item for 250 ms, a timing schedule that maps on to that used in several existing studies examining value-based prioritization in WM (e.g., Hitch et al., 2018; Hu et al., 2014, 2016). Other WM studies have used longer exposure times, typically 500 ms per item (e.g., Allen et al. 2021; Atkinson et al., 2018, 2019; Hu et al., 2023; Sandry et al., 2014, 2020; Sandry & Ricker, 2020). Although value effects at WM appear to be unaffected by increased encoding time (Allen et al., 2021), it is possible that longer encoding times may result in more durable prioritization effects, which are then more likely to persist into LTM. Indeed, Sandry et al. (2020) observed some effects of WM-allocated value on LTM when presenting items for 500 ms each during the WM phase. To examine this, Experiment 2 implemented an encoding time of 500 ms per item during the WM phase, while all other methodological details were unchanged from Experiment 1. We were interested in whether the effects of value-based prioritization on WM would now reliably extend to the surprise LTM test.

### Method

#### Participants

Thirty participants completed the experiment (*M. age* = 20.27 years, *SD* = 2.42 years; range = 18–29 years; three males). Participants were fluent English speakers, had normal or corrected-to-normal vision, and had no known learning difficulties. They were reimbursed with course credit or cash. The study was approved by the School of Psychology Ethics Committee at the University of Leeds (Ethics reference number 17-0017).

#### Design, materials and procedure

Methodology was closely based on Experiment 1, with the key difference being that stimuli were presented for 500 ms per item in the WM phase.

#### Data analysis

As in Experiment 1, participants who anticipated the second memory task were excluded from all analysis. This resulted in six out of 30 participants (20%) being excluded. Analysis was therefore conducted on the data for 24 participants. Generally, these participants did not report thinking about the objects much in the interval between the WM and LTM tests (*M* = 2.04, *SE* = 0.34, where 1 = not at all and 7 = all of the time).

RT processing was the same as in Experiment 1. In this experiment, 385/2304 trials in the WM phase (16.71%) and 2,087/4,608 in the LTM phase (45.29%) were discarded due to participants responding incorrectly. RTs over 20,000 ms (20 s) were then excluded, followed by RTs that fell 2.5 SDs above or below the mean for each condition for each participant. These steps resulted in the removal of 20/2,304 data points in the WM phase (0.87%) and 7/4,608 data points in the LTM phase (0.15%).

### Results

#### Working memory

##### Accuracy

**Fig. 5:**
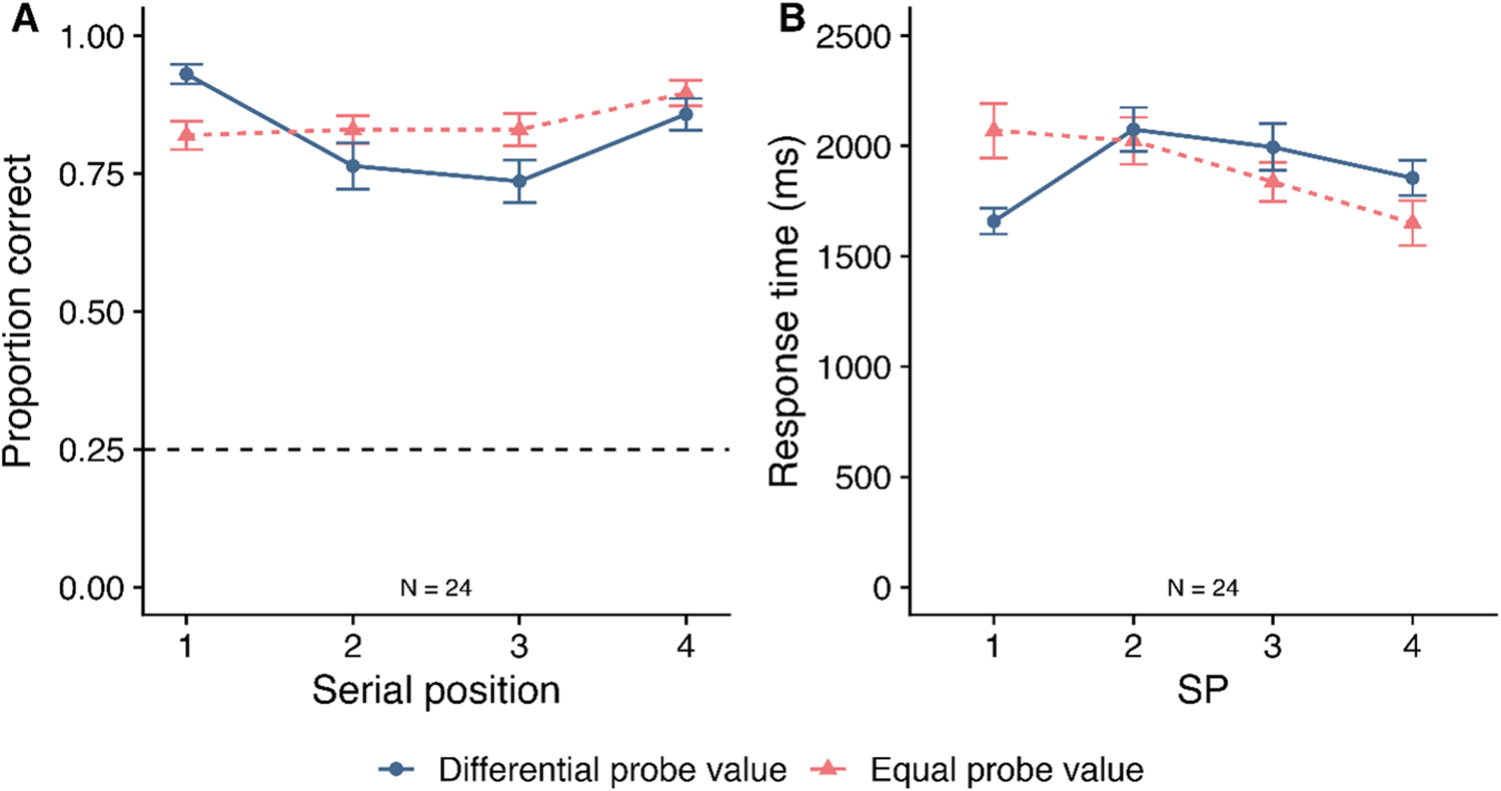
Mean proportion correct (**Panel A**) and mean response time (**Panel B**) in the working memory task in Experiment 2, a function of probe value and serial position (SP). Error bars display SE. The dashed grey line at 0.25 in Panel A reflects chance rate

Proportion correct in the WM task is displayed in Fig. 5A as a function of probe value and SP. A 2 (Probe value: differential vs. equal) × 4 (SP: 1–4) within-subjects ANOVA was conducted. No significant effect of probe value emerged (*F*(1, 23) = 1.28, *MSE* = 0.02, *p* = .270*,* ${\eta }_{p}^{2}$ = .05; *BF*_*10*_ = 0.29, *BF*_*01*_ = 3.42), although there was a main effect of SP (*F*(3, 69) = 7.02, *MSE* = 0.02, *p* < .001*,* ${\eta }_{p}^{2}$ = .23; *BF*_*10*_ = 147.96). Bonferroni-Holm post hoc comparisons revealed significant differences between SP1 (*M* = .88, *SE* = .02) and SP2 (*M* = .80, *SE* = .02; *p* = .010; *BF*_*10*_ = 3.18), SP1 and SP3 (*M* = .79, *SE* = .03; *p* = .008; *BF*_*10*_ = 9.07), SP2 and SP4 (*M* = .88, *SE* = .02; *p* = .036; *BF*_*10*_ = 5.79), and SP3 and SP4 (*p* = .036; *BF*_*10*_ = 9.82). A significant interaction between probe value and SP also emerged (*F*(3, 69) = 5.85, *MSE* = 0.02, *p* = .001*,* ${\eta }_{p}^{2}$ = .20; *BF*_*10*_ = 45.62). BF analysis revealed that the best model included a main effect of SP, as well as an interaction between probe value and SP (*BF*_*10*_ = 3145.70 relative to a null model containing participant only).

To investigate the interaction further, a series of paired-sample t-tests were conducted to investigate if an effect of probe value was observed at each SP. There was a significant effect at SP1 (*t*(23) = 3.52, *p* = .007, *d* = 0.72; *BF*_*10*_ = 20.51), with participants recognising more items correctly in the differential probe value condition (*M* = .93, *SE* = .02) relative to the equal probe value condition (*M* = .82, *SE* = .03). There were no significant effects at the other SPs (*t* ≥ -2.21 and ≤ -1.35, *p* ≥ .111, *d* ≥ -0.45 and ≤ -0.28; *BF*_*10*_ ≥ 0.48 and ≤ 1.65, *BF*_*01*_ ≥ 0.61 and ≤ 2.09).

##### Response times

Mean RT in the WM phase (and SE) is displayed in Fig. 5B as a function of probe value and SP. A 2 (Probe value: differential vs. equal) × 4 (SP: 1–4) within-subjects ANOVA revealed no main effect of probe value (*F*(1, 23) < .01, *MSE* = 157,734.57, *p* = .993*,* ${\eta }_{p}^{2}$ < .01; *BF*_*10*_ = 0.16; *BF*_*01*_ = 6.41). A main effect of SP emerged (*F*(3, 69) = 9.46, *MSE* = 76,733.88, *p* < .001*,* ${\eta }_{p}^{2}$ = .29; *BF*_*10*_ = 368.62). Bonferroni-Holm-corrected pairwise comparisons revealed a significant difference between SP1 (*M* = 1864, *SE* = 80.9) and SP2 (*M* = 2049, *SE* = 92.6; *p* = .006; *BF*_*10*_ = 11.11), SP2 and SP3 (*M* = 1916, *SE* = 88.5; *p* = .047; *BF*_*10*_ = 3.05), SP2 and SP4 (*M* = 1753, *SE* = 79.5; *p* < .001; *BF*_*10*_ = 1880.99), and SP3 and SP4 (*M* = 1753, *SE* = 79.5; *p* = .009; *BF*_*10*_ = 7.26). There was an interaction between probe value and SP (*F*(3, 69) = 11.33, *MSE* = 84074.13, *p* < .001*,* ${\eta }_{p}^{2}$ = .33; *BF*_*10*_ = 7073.45). The BF analysis revealed that the best model included a main effect of SP, and the probe value and SP interaction (*BF*_*10*_ > 10,000 relative to a null model containing participant only).

To investigate the probe value and SP interaction, Bonferroni-Holm-corrected paired-sample t-tests were conducted to examine whether a significant effect of probe value emerged at each SP. There was a significant effect at SP1 (*t*(23) = -3.90, *p* = .003, *d* = -0.80; *BF*_*10*_ = 47.14), with faster responses in the differential-value condition (*M* = 1659, *SE* = 58.3) relative to the equal probe value condition (*M* = 2070, *SE* = 123.5). The difference also approached significance at SP4 (*t*(23) = 2.30, *p* = .092, *d* = 0.47; *BF*_*10*_ = 1.91), with numerically faster responses in the equal probe value (*M* = 1651, *SE* = 101.4) condition than the differential probe value condition (*M* = 1855, *SE* = 79.3). No significant difference emerged at SP2 (*t*(23) = 0.56, *p* = .579, *d* = 0.12; *BF*_*10*_ = 0.25, *BF*_*01*_ = 4.03) or SP3 (*t*(23) = 1.90, *p* = .141, *d* = 0.39; *BF*_*10*_ = 0.99; *BF*_*01*_ = 1.01).

#### Long-term memory

##### Accuracy

**Fig. 6:**
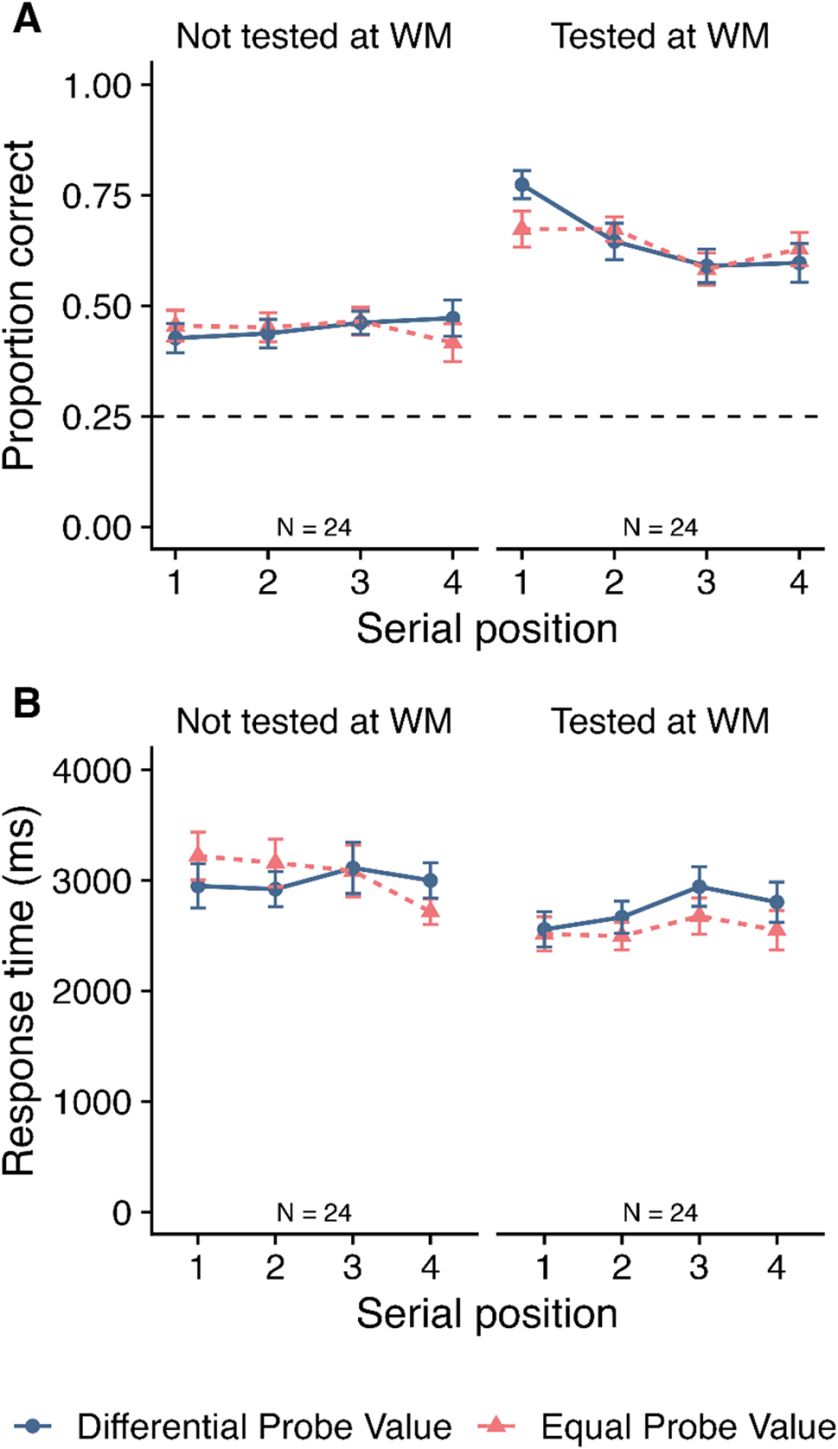
Mean proportion correct (**Panel A**) and mean response time (**Panel B**) in the long-term memory phase of Experiment 2 as a function of probe value, serial position (SP) and tested-at-working memory (WM). Error bars show SE. The dashed grey line at 0.25 in Panel A reflects chance rate

Proportion correct as a function of probe value, SP, and tested-at-WM is displayed in Fig. 6A. A 2 (probe value: differential vs. equal) × 4 (SP: 1–4) × 2 (tested-at-WM: tested vs. not tested) within-subjects ANOVA was conducted. This revealed no main effect of probe value (*F*(1, 23) = 0.22, *MSE* = .02, *p* = .646*,* ${\eta }_{p}^{2}$ = .01; *BF*_*10*_ = 0.13, *BF*_*01*_ = 7.78). There was also no significant main effect of SP, although this approached significance (*F*(3, 69) = 2.49, *MSE* = .03, *p* = .068*,* ${\eta }_{p}^{2}$ = .10; *BF*_*10*_ = 0.77, *BF*_*01*_ = 1.29). There was a main effect of tested-at-WM (*F*(1, 23) = 122.25, *MSE* = .03, *p* < .001*,* ${\eta }_{p}^{2}$ = .84; *BF*_*10*_ > 10,000), with higher accuracy for items that were tested (*M* = .65, *SE* = .03) relative to items that were not tested (*M* = .45, *SE* = .02). There was no interaction between probe value and SP (*GG corrected F*(2.05, 47.25) = .70, *MSE* = .03, *p* = .504*,* ${\eta }_{p}^{2}$ = .03; *BF*_*10*_ = 0.06, *BF*_*01*_ = 17.10), or probe value and tested-at-WM (*F*(1, 23) = .23, *MSE* = .01, *p* = .635*,* ${\eta }_{p}^{2}$ = .01; *BF*_*10*_ = 0.16, *BF*_*01*_ = 6.09). However, a significant interaction emerged between SP and tested-at-WM (*F*(3, 69) = 5.66, *MSE* = .02, *p* = .002*,* ${\eta }_{p}^{2}$ = .20; *BF*_*10*_ = 26.46; see OSM for post hoc tests). There was also a significant three-way interaction between probe value, SP and tested-at-WM (*F*(3, 69) = 3.22, *MSE* = .01, *p* = .028*,* ${\eta }_{p}^{2}$ = .12), although the BF analysis was slightly in favour of no effect (*BF*_*10*_ = 0.77, *BF*_*01*_ = 1.31). The BF analysis indicated that the best model included a main effect of tested-at-WM, as well as an interaction between SP and tested-at-WM (*BF*_*10*_ > 10,000 relative to null model containing participant only).

To explore the significant three-way interaction between probe value, SP and tested-at-WM, two separate 2 (Probe value: differential vs. equal) × 4 (SP: 1–4) within-subject ANOVAs were conducted on the data from the tested at WM condition and the not tested at WM condition. When the items were not tested at WM, there were no main effects and no interaction (*F* ≤ 0.82, *p* ≥ .487, *BF*_*10*_ ≤ 0.16, *BF*_*01*_ ≥ 6.37). When the items were tested at WM, there was no main effect of probe value (*F*(1, 23) = 0.50, *MSE* = 0.01, *p* = .488*,* ${\eta }_{p}^{2}$ = .02; *BF*_*10*_ = 0.19, *BF*_*01*_ = 5.29), although there was a main effect of SP (*F*(3, 69) = 8.18, *MSE* = .02, *p* < .001*,* ${\eta }_{p}^{2}$ = .26; *BF*_*10*_ = 4796.45). Bonferroni-Holm pairwise comparisons revealed that SP1 (*M* = .72, *SE* = .03) significantly differed from SP3 (*M* = .59, *SE* = .03; *p* < .001; *BF*_*10*_ = 1454.73) and SP4 (*M* = .61, *SE* = .04; *p* = .007; *BF*_*10*_ = 93.58). A significant difference also emerged between SP2 (*M* = .66, *SE* = .03) and SP3 (*p* = .033; *BF*_*10*_ = 17.30). Additionally, there was a marginally significant interaction between probe value and SP (*GG corrected F*(2.32, 53.29) = 3.13, *MSE* = .02, *p* = .045*,* ${\eta }_{p}^{2}$ = .12), although the BF was entirely equivocal (*BF*_*10*_ = 1.00). To investigate this interaction, Bonferroni-Holm-corrected paired-sample t-tests were conducted to investigate whether a probe value effect emerged at each SP. A significant effect emerged at SP1 (*t*(23) = 2.84, *p* = .037, *d* = 0.58; *BF*_*10*_ = 5.15), with higher accuracy in the differential probe value condition (*M* = .77, *SE* = .03) than in the equal probe value condition (*M* = .67, *SE* = .04). No significant effects emerged at the other SPs (*t* ≥ -1.16 and ≤ 0.17, *p* ≥ .773, *d* ≥ -0.24 and ≤ 0.04; *BF*_*10*_ ≤ 0.39, *BF*_*01*_ ≥ 2.56).

##### Trials correct at working memory

The LTM boost for high-value items for items that had been tested at WM might have not resulted from a durable probe value boost per se (Reaves et al., 2016). Instead, it may have been driven by a carry-over effect from testing at WM, with participants recognising more of the items at SP1 in the differential probe value condition relative to the equal probe value condition at this phase. To investigate this, the LTM data were re-analysed including only trials that were tested at WM and on which participants responded correctly (Reaves et al., 2016). This was conducted to investigate whether items initially associated with a higher value were still more likely to be recognised at LTM when performance at WM was controlled for.

**Fig. 7:**
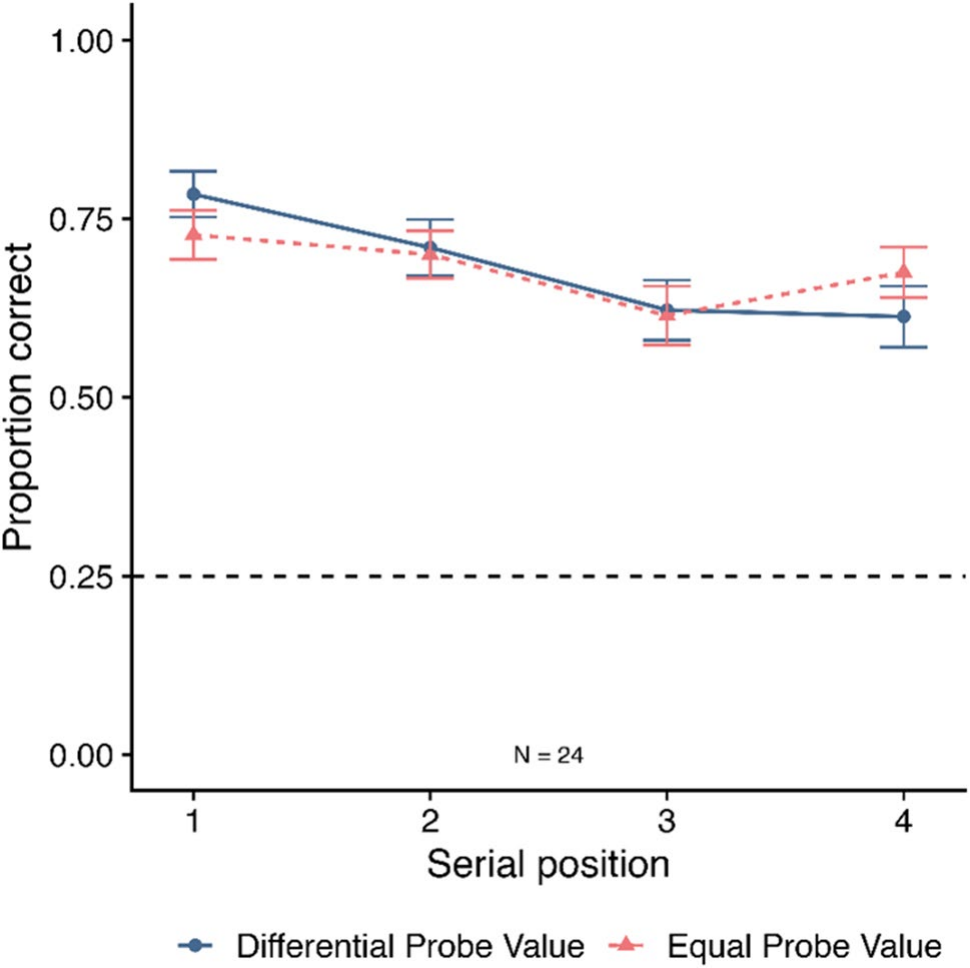
Proportion correct at long-term memory in Experiment 2 for trials that participants were tested on at working memory and responded correctly, as a function of probe value and serial position. Error bars denote SE. The dashed grey line at 0.25 displays chance rate

Only the condition where items were tested and responded to correctly at WM were included in this analysis. Thus, 385/2304 trials were removed (16.71%) due to participants responding inaccurately at WM. Proportion correct at LTM for the remaining trials is displayed in Fig. 7, as a function of probe value and SP. A 2 (probe value: differential vs. equal) × 4 (SP: 1–4) repeated-measures ANOVA revealed no significant main effect of probe value (*F*(1, 23) = 0.02, *MSE* = .02, *p* = .882*,* ${\eta }_{p}^{2}$ < .01; *BF*_*10*_ = 0.16, *BF*_*01*_ = 6.35), although there was a main effect of SP (*F*(3, 69) = 7.33, *MSE* = .03, *p* < .001*,* ${\eta }_{p}^{2}$ = .24; *BF*_*10*_ = 1025.32). Pairwise comparisons revealed higher accuracy at SP1 (*M* = .76, *SE* = .03) relative to SP3 (*M* = .62, *SE* = .03; *p* = .002) and SP4 (*M* = .64, *SE* = .03; *p* = .003). The interaction between probe value and SP was not significant (*F(*3, 69) = 1.62, *MSE* = .02, *p* = .192*,* ${\eta }_{p}^{2}$ = .07), with the BF analysis providing evidence of no effect (*BF*_*10*_ = 0.25, *BF*_*01*_ = 4.01). The BF analysis revealed that the best model included a main effect of SP (*BF*_*10*_ = 858.68 relative to a null model containing participant only). From this, it can be concluded that there was no significant probe value boost at LTM when only the trials participants answered correctly at WM were considered. This suggests that the probe value boost at LTM when items were tested at WM may reflect a carry-over effect from participants being more likely to respond correctly at WM.

##### Response times 

Mean RT (and SE) in the LTM test is displayed in Fig. 6B as a function of probe value, SP and tested-at-WM. A 2 (Probe value: differential vs. equal) × 4 (SP: 1–4) × 2 (Tested-at-WM: tested vs. not tested) repeated-measures ANOVA revealed no main effect of probe value (*F*(1, 23) = 0.68, *MSE* = 630,552.25, *p* = .419*,* ${\eta }_{p}^{2}$ = .03; *BF*_*10*_ = 0.23, *BF*_*01*_ = 4.32) or SP (*F*(3, 69) = 2.16, *MSE* = 298,793.14, *p* = .101*,* ${\eta }_{p}^{2}$ = .09; *BF*_*10*_ = 0.19, *BF*_*01*_ = 5.37). A main effect of tested-at-WM emerged (*F*(1, 23) = 42.38, *MSE* = 309,338.61, *p* < .001*,* ${\eta }_{p}^{2}$ = .65; *BF*_*10*_ > 10,000), with faster performance in the tested condition (*M* = 2651, *SE* = 136) relative to the not tested condition (*M* = 3021, *SE* = 157). There was a significant interaction between probe value and SP (*F*(3, 69) = 3.09, *MSE* = 233,980.55, *p* = .033*,* ${\eta }_{p}^{2}$ = .12), although the BF analysis provided anecdotal evidence of no effect (*BF*_*10*_ = 0.55, *BF*_*01*_ = 1.81). The frequentist analysis also revealed a significant interaction between probe value and tested-at-WM (*F*(1, 23) = 4.73, *MSE* = 276,021.56, *p* = .040*,* ${\eta }_{p}^{2}$ = .17, *BF*_*10*_ = 1.29). No other significant interactions emerged (*F* ≤ 1.96, *p* ≥ .128, *BF*_*10*_ ≤ 0.42, *BF*_*01*_ ≥ 2.38). The BF analysis revealed the best model contained a main effect of tested-at-WM and an interaction between probe value and tested-at-WM (*BF*_*10*_ > 10,000 relative to the null model containing participant only).

To investigate the interaction between probe value and SP, four paired-samples t-tests were conducted to investigate whether an effect of probe value emerged at each SP, averaging over WM test. After correction, no significant effects emerged (*t* ≥ -1.08 and ≤ 2.17, *p* ≥ .163, *d* ≥ -0.22 and ≤ 0.44). The BF analysis revealed anecdotal evidence for an effect at SP4 (*BF*_*10*_ = 1.53), but there was no evidence in favour of an effect the other SPs (*BF*_*10*_ ≤ 0.40, *BF*_*01*_ ≥ 2.50).

To investigate the interaction between probe value and tested-at-WM, t-tests were conducted to investigate whether an effect of probe value emerged in the tested and not tested conditions, averaging over SP. No probe value effect emerged, both when the items were tested at WM (*t*(23) = 2.21, *p* = .074, *d* = 0.45, *BF*_*10*_ = 1.65) and when they were not tested at WM (*t*(23) = -0.45, *p* = .654, *d* = -0.09, *BF*_*10*_ = 0.24, *BF*_*01*_ = 4.24).

### Discussion

In line with Experiment 1 and previous findings (e.g., Atkinson et al., 2018, 2022; Hitch et al., 2018; Hu et al., 2014; Sandry et al., 2020), WM accuracy was improved for the high-value item. This confirms that such effects are reliable when using recognition of real images. An RT effect at WM was also observed (Sandry & Ricker, 2020; Sandry et al., 2020), with participants responding more quickly at SP1 in the differential-value condition relative to the equal-value condition. In this experiment, there were no costs to less valuable items, although performance was numerically higher in the equal-value condition at SP2, SP3 and SP4 (see Fig. 5A).

At LTM, the findings were more mixed. In line with Experiment 1, there was a large testing effect, likely driven by a combination of the item being tested and re-presented during the WM test phase. Regarding the prioritization effect, the results were slightly different. In line with Experiment 1, the evidence indicated no interaction between probe value and SP. The BF analysis also found no support for the three-way interaction between value, SP and tested-at-WM. However, this three-way interaction was significant in the frequentist analysis. Further analysis revealed a small but significant prioritization effect at SP1 during the LTM phase when the item was tested at WM, but no such effect when the item had not been tested at WM. Given that a prioritization effect emerged at WM, one possibility is that the effect at LTM might reflect a carry-over effect due to participants being more likely to respond correctly at WM (Reaves et al., 2016). To examine this possibility, we re-analysed the data including only trials where participants were tested on the item at WM and responded correctly. In this analysis, no significant prioritization effect was observed at LTM, suggesting that the effect may indeed reflect a carry-over effect from participants being more likely to respond correctly to the item at WM.

## Cross-experimental analyses

Given the similarity between Experiments 1 and 2, cross-experimental analyses were conducted to rule out the possibility that the lack of significant effects at LTM are due to a lack of statistical power. This sample size (N = 58) is more in line with Sandry et al. (2020; *N* = 67 in Experiment 1 post-exclusions), who found an effect of prioritization at WM on a LTM test. To maximise power, the effect of presentation time (which differed between experiments) was ignored. At WM, a 2 (probe value) × 4 (SP) within-subjects ANOVA was conducted. At LTM, a 2 (probe value) × 4 (SP) × 2 (tested-at-WM) within-subjects ANOVA was conducted. The full results are presented in the OSM. At WM, there was a significant effect of probe value overall, indicating overall higher accuracy in the equal probe value condition. There was, however, a significant interaction between probe value and SP. This was driven by significantly higher accuracy at SP1 in the differential probe value condition, but the reverse pattern at all other SPs. Moreover, RTs were faster at SP1 in the differential probe value condition. An RT cost was observed at SP3 and SP4. At LTM, there was a significant effect of tested-at-WM on accuracy and RT, but no significant effect of probe value, and no interactions including probe value. To summarise, this indicates a significant effect of value at WM, with participants responding more accurately and faster to the high-value item. This is accompanied by accuracy costs at all items, as well as RT costs at most items. At LTM, there was no evidence of a value effect.

## General discussion

Two experiments investigated whether prioritizing an item for a WM test also results in boosts on a surprise LTM test. We examined this question in varying task contexts, including whether the item was tested or not at WM (Experiments 1 and 2), and across shorter (Experiment 1; 250 ms) and longer (Experiment 2; 500 ms) presentation times. At WM, participants responded more accurately at the first SP when it was associated with a higher value relative to a condition in which all items were equally valuable. This is in line with a large body of work showing that value-based prioritization enhances performance at WM (e.g., Atkinson et al., 2018, 2022; Hitch et al., 2018, 2020; Hu et al., 2014; Sandry et al., 2020). Nearly all this prior research has used simple coloured shapes as to-be-remembered material, and tested WM using cued recall. The present study extends these effects to the use of real-world images and a recognition task. There was also some evidence that participants responded faster to SP1 in the differential-value condition relative to when all items were equally valuable (Experiment 2) (Sandry et al., 2020). Some evidence of costs to less valuable items was also apparent, which was particularly clear with increased statistical power provided by the cross-experimental analyses. This is in line with previous research, indicating that a boost to the high-value item results in a resource trade-off, whereby lower-value items are remembered less accurately (e.g., Atkinson et al., 2018; Brissenden et al., 2023; Hu et al., 2014; Sandry et al., 2020).

Turning to LTM, with shorter presentation times (250 ms per item; Experiment 1), prioritizing an item for WM did not impact on performance on the surprise LTM test, regardless of whether the item had been tested at WM or not. With longer presentation times (500 ms per item; Experiment 2), there was some possible evidence that prioritizing an item at WM enhanced performance at LTM, but only when the item had been tested at WM. On the face of it, this would suggest that WM prioritization can benefit LTM when more time is available during encoding, and this item is then drawn on at the immediate test. However, we would exercise caution in attaching any strong interpretative weight to this finding for several reasons. Firstly, evidence for the interaction was not supported by the BF analysis, which provided evidence slightly in favour of no interaction. Secondly, the effect was not present when limiting the analysis to items that were correctly recognised at WM, suggesting that any effect may result from a carry-over effect of participants being more likely to respond correctly for high-value items at WM. Thirdly, the effect did not emerge when cross-experimental analyses were conducted. As such, these experiments provide no consistent evidence that prioritizing an item for a WM test influences LTM recognition. In contrast, participants responded both more accurately and faster at LTM for items that featured in the WM test phase, in both experiments.

Evidence that prioritizing particularly valuable information in WM does not consistently enhance LTM performance is broadly in line with Jeanneret et al. (2023), who found no significant effects of reward-based prioritization on WM at LTM. Thus, there seems to be little evidence that prioritising information in a visual WM task enhances LTM, regardless of whether information is presented sequentially (current study) or simultaneously (Jeanneret et al., 2023). In contrast, Sandry et al. (2020) observed significant value effects at WM on LTM performance, but only in the condition where items had not been tested at WM. Why might this difference in findings have emerged? One set of possibilities may be derived from methodological differences between studies. For example, differing outcomes may reflect the type of to-be-remembered material. Whilst the current study and Jeanneret et al. (2023) used visual images, Sandry et al. (2020) used visually presented words. Within WM, prioritization effects have been observed across a wide range of material (e.g., Atkinson et al., 2018, 2021; Hu et al., 2016; Johnson & Allen, 2023; Roe et al., 2024; Sandry & Ricker, 2020; Sandry et al., 2014). However, it may be that the durability of such effects differs depending on modality.

A second possibility is that the difference in findings may reflect the type of LTM test. The current study and Jeanneret et al. (2023) used recognition tests at LTM, whilst Sandry et al. (2020) used a free-recall test. Recall relies on recollection of information, whereas recognition can reflect both recollection and familiarity. If participants can rely on familiarity for low-value or equal-value items, this may add noise and reduce the size of any value effect at LTM. Indeed, manipulations such as the testing effect have been found to have larger impacts when the final test employs free recall rather than recognition (Rowland, 2014). As such, it may be that value effects do persist into LTM to some extent but are only reliably detectable with recall tasks. Further research is needed to explore these possibilities, which would provide important insights into the boundary conditions concerning the durability of value-based prioritization effects in WM.

Another related question is whether the effect of value at WM on LTM differs depending on which serial position is more valuable. Within WM, the effect of value appears to be robust across SPs, with effects observed at the first position (e.g., Atkinson et al., 2018; Hu et al., 2014), but also at middle and final serial positions (Atkinson et al., 2021; Hu et al., 2014, 2023; Hitch et al., 2018; Hu et al., 2023). However, effect durability may in part reflect initial position in the originally encoded sequence. There is perhaps some tentative evidence of this in Sandry et al. (2020, Fig. 4), with the effect appearing to be at least numerically larger when participants were encouraged to prioritize the final serial position at WM, relative to when they were encouraged to prioritize earlier serial positions. This possibility is purely speculative at present, and systematic examination of any serial position effects on long-term value persistence would be required before any firm claims can be made.

As outlined in the *Introduction*, previous research investigating prioritization in WM has compared high-value items in one condition either to the same serial position in a different condition in which all items are equally valuable (e.g., Atkinson et al., 2018, 2021; Sandry et al., 2014, 2020) or to an item in the same condition that has a lower value (e.g., Hu et al., 2014; Hu et al., 2016). The present study and Sandry et al. (2020) have adopted the former approach, comparing high-value items to equal-value items. This approach reflects only the boost obtained from an item being of high value. It represents a somewhat stricter comparison than comparing high- and low-value items, which reflects both the boost to high-value items and the cost to low-value items. Effects that last into LTM may be more likely to be reliably observed when comparing high- and low-value information, though by itself this is not a sufficient explanation given that Jeanneret et al. (2023) found null effects at LTM when employing this approach.

Evidence that prioritizing an item for a WM test does not consistently affect LTM somewhat contrasts with research indicating that prioritizing an item for a LTM test enhances delayed retrieval (e.g., Adcock et al., 2006; Castel et al., 2002, 2011; Gruber & Otten, 2010; Murty & Adcock, 2014; Shigemune et al., 2010; Wittmann et al., 2005; Yin et al., 2021). This may suggest that although prioritizing an item for a WM test has only limited effects on a surprise LTM test, prioritizing an item in preparation for a later test can result in durable and long-lasting effects. There are, however, several important task differences between studies which have informed participants of the LTM test (e.g., Adcock et al., 2006; Castel et al., 2002; Gruber & Otten, 2010; Murty & Adcock, 2014) and those that have not (e.g., the current study; Jeanneret et al., 2023; Sandry et al., 2020). For example, studies that have informed participants of the LTM test have often (but not always) used more meaningful rewards (e.g., monetary rewards vs. notional points used in the current study; Jeanneret et al., 2023; Sandry et al., 2020). As such, further research is therefore needed to examine the impact that awareness of the final test has on the durability of the value effect.

Evidence that prioritizing an item for a WM test did not enhance LTM also somewhat contrasts with some findings from the cueing literature showing that cueing an item at WM enhances LTM (e.g., Reaves et al., 2016; Strunk et al., 2018). This may reflect the extent to which the attentional manipulation predicts which item is likely to be tested (Jeanneret et al., 2023). For instance, whilst cueing studies typically use 100% valid cues (whereby the cue always identifies which item will be tested at WM; e.g., Jeanneret et al., 2023; Reaves et al., 2016; Strunk et al., 2018), value information does not typically inform participants which item will, or is likely to be, tested. This may result in different approaches; at the point at which the retro-cue is presented, participants may reduce the memory load to one item (Souza & Oberauer, 2016). In contrast, given that value information does not predict which item will be assessed, it would not be beneficial to abandon the other items. This may impact upon the extent to which the targeted item (e.g., cued or prioritized based on item value) is actively maintained during the retention interval. As increased WM maintenance of a particular stimulus is associated with retrieval at LTM (Hartshorne & Makovski, 2019), this may explain why retro-cue effects are observed at LTM, whilst value-based prioritization effects are not consistently observed (Jeanneret et al., 2023; Sandry et al., 2020).

Although not of primary interest, a testing effect was observed, whereby items that were tested at WM were recognised more accurately and faster at LTM than items that were not assessed previously. This is in line with previous research that has found that testing information enhances later memory (e.g., Kang et al., 2007; Nungester & Duchastel, 1982; Rowland, 2014). However, it is important to note that the items that had been tested at WM had been viewed twice by participants (i.e., during encoding and during the WM test phase), whereas items that had not been tested at WM had only been presented once (i.e., during encoding). The ‘testing effect’ observed is therefore likely to result from a combination of a retrieval effect and the additional presentation during the WM retrieval phase.

More generally, the current experiments add to a growing body of literature indicating the conditions in which processes at WM do, and do not, enhance longer-term retention. Evidence that items that were tested at WM enhance longer-term retention is in line with a growing number of studies indicating that processes that boost WM performance can enhance LTM performance (Camos & Portrat, 2015; Cotton & Ricker, 2021). Conversely, evidence that value-based prioritization at WM does not consistently enhance LTM performance demonstrates that processes that enhance WM do not always result in durable boosts that enhance longer-term retention (Camos & Portrat, 2015; Overkott & Souza, 2022). It would be fruitful for future research to further elucidate the conditions in which processes at WM do, and do not, impact upon LTM performance.

To summarise, across both experiments, we found clear evidence that individuals can prioritize particularly valuable information at WM with resulting impacts on recognition response accuracy and speed. However, we found only very limited evidence of automatic continuation into LTM, with any observed effect dependent on the item originally being tested in the WM phase. Thus, the benefits of attentional prioritization in WM can be somewhat ephemeral and do not necessarily translate into longer-term performance. Further research is therefore needed to elucidate the boundary conditions in which directing attention in WM does, and does not, enhance long-term retention.

## Supplementary Information

Below is the link to the electronic supplementary material.

Supplementary file1 (DOCX 858 KB)
