# Supplementary material for: Does value-based prioritization at working memory enhance long-term memory?
Source: Mem Cognit. 2024 Feb 20;52(8):1983–98. doi: 10.3758/s13421-024-01532-9 (PMC11588910; doi:10.3758/s13421-024-01532-9)
Supplement: Supplementary file 1 — Supplementary file1 (DOCX 858 KB) [file 13421_2024_1532_MOESM1_ESM.docx]

**Supplementary materials for ‘Does value-based prioritization at working memory enhance long-term memory’? Atkinson, Waterman, & Allen.**

1. Description of the filler tasks
2. Questionnaire used in Experiment 1 and 2
3. Experiment 1 further analysis: Breaking down interactions
4. Experiment 1 further analysis: Questionnaire
5. Experiment 2 further analysis: Breaking down interactions
6. Experiment 2 further analysis: Questionnaire
7. Cross-experimental analyses
8. **Description of the filler tasks**

Following the WM phase, participants completed a series of filler tasks. Three WM tests were used in order to reduce the likelihood that participants would anticipate the LTM test: FDR, BDR, and the Corsi blocks tapping task. In the FDR task, participants were read out series of numbers by the experimenter, which they had to repeat back in the same order. Participants first completed two practise trials, which contained two digits per sequence (e.g. 3-5). Following this, participants were presented with sequences that slowly increased in length from two digits at the start of the task to nine digits at the end of the task. Three trials were given at each length. If participants successfully recalled two or three of the sequences at a given length, they progressed onto the next length. If they answered 0 or 1 correct, the task ended. The BDR task used similar sequences, with participants asked to recall the digits in a backwards order (e.g. 3-5-1 should be recalled as 1-5-3). In the Corsi blocks tapping task, participants were presented with a Lego board containing nine squares arranged in a random order. These squares contained numbers on them in order to make it easier to administrate the task, but these were only visible to the experimenter. The experimenter tapped a pattern, which the participant was asked to reproduce in the same order. As with the digit recall tasks, participants first completed two practise trials. The sequence length gradually increased from two to nine, although the task ended if participants responded correctly on zero or one trials at a given length. The order of these tasks was fully counterbalanced across participants. The tasks took approximately 10 minutes in total.

1. **Questionnaire used in Experiment 1 and 2**

*Please answer this questionnaire honestly. Your responses to this questionnaire will not affect the reward you receive for participating in the study.*

1. Did you expect to be asked about the objects again after the working memory task (when you were shown the objects/tested on them the first time)?

Yes No

1. If yes, why?

1. To what extent did you think about the objects between encoding (when you were shown the objects/tested on them the first time) and the final test? (1 = not at all; 7 = all of the time).

**
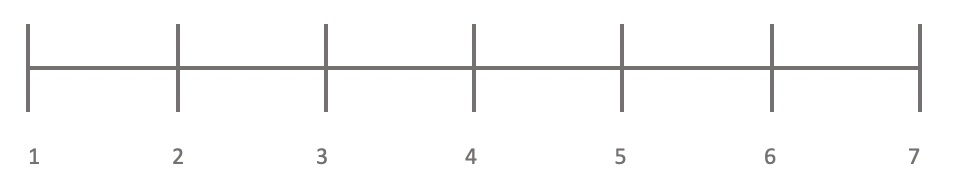
**

1. Were you thinking about any objects in particular during the interval between encoding (when you were shown the objects/tested on them the first time) and the final test? If so, which ones?

**Initial task:**

**The following questions refer to the test immediately after encoding:**

How do you think trying to prioritise the 1^st^ position affected your memory **for this item** compared to the condition in which every object was worth 1 point? (1 = large negative effect, 5 = no effect, 9 = large positive effect)

| 1 | 2 | 3 | 4 | 5 | 6 | 7 | 8 | 9 |
| --- | --- | --- | --- | --- | --- | --- | --- | --- |

How do you think trying to prioritise the 1^st^ position affected your memory for **the less valuable items** compared to the condition in which every object was worth 1 point? (1 = large negative effect, 5 = no effect, 9 = large positive effect)

| 1 | 2 | 3 | 4 | 5 | 6 | 7 | 8 | 9 |
| --- | --- | --- | --- | --- | --- | --- | --- | --- |

**Final task:**

**The following questions refer to the final test of the items you just completed:**

How you think prioritising the 1^st^ position during the initial task affected your memory for **these items** during the final test relative to the condition in which all items were worth 1 point? (1 = large negative effect, 5 = no effect, 9 = large positive effect)

| 1 | 2 | 3 | 4 | 5 | 6 | 7 | 8 | 9 |
| --- | --- | --- | --- | --- | --- | --- | --- | --- |

How you think prioritising the 1^st^ position during the initial task affected your memory for **the less valuable items** during the final test relative to the condition in which all items were worth 1 point? (1 = large negative effect, 5 = no effect, 9 = large positive effect)

| 1 | 2 | 3 | 4 | 5 | 6 | 7 | 8 | 9 |
| --- | --- | --- | --- | --- | --- | --- | --- | --- |

**Do you have any further questions about the experiment?**

1. **Experiment 1 further analysis: Breaking down interactions**

Accuracy: Long-term memory

*SP and tested-at-WM interaction*: When the item had not been tested at WM, there was no significant effect of SP (*F*(3, 99) = 1.09, *MSE* = 0.01, *p* = .355*,* $\eta_{p}^{2}$ = .03, *BF_10_* = 0.14, *BF_01_* = 7.15). In contrast, when items that had been tested at WM, there was a significant effect of SP (*F*(3, 99) = 2.97, *MSE* = 0.01, *p* = .036*,* $\eta_{p}^{2}$ = .08, *BF_10_* = 1.16). Post-hoc tests revealed no significant differences after correction, although the difference between SP1 (*M* = 0.63; *SE* = 0.02) and SP3 (*M* = 0.57, *SE* = 0.03) approached significance (*p* = .055; *BF_10_* = 4.62).

1. **Experiment 1 further analysis: questionnaire**

| Mean perceived effect (and SE) of prioritization at SP1 (where the manipulation was targeted) and at other SPs at WM and LTM. Possible values are between -4 and +4, where 0 = no effect, positive values reflect a perceived positive effect, and negative values reflect a perceived negative effect. | | |
| --- | --- | --- |
|  | Effect at SP1 (where manipulation was targeted) | Effect at other SPs |
| WM | 1.12 (0.33) | -1.56 (0.29) |
| LTM | 1.09 (0.32) | -1.62 (0.28) |

1. **Experiment 2 further analysis: Breaking down interactions**

Accuracy: Long-term memory

*SP and tested-at-WM interaction*: When the item had not been tested at WM, there was no significant effect of SP (*F*(3, 69) = 0.20, *MSE* = 0.01, *p* = .897*,* $\eta_{p}^{2}$ = .01, *BF_10_* = 0.07, *BF_01_* = 14.09). In contrast, when items that had been tested at WM, there was a significant effect of SP (*F*(3, 69) = 8.18, *MSE* = 0.01, *p* < .001*,* $\eta_{p}^{2}$ = .26, *BF_10_* = 247.75). Post-hoc tests revealed significant differences between SP1 (*M* = 0.72; *SE* = 0.03) and SP3 (*M* = 0.59, *SE* = 0.03, *p* < .001, *BF_10_* = 541.01), SP1 and SP4 (*M* = 0.61, *SE* = 0.04, *p* = .007, *BF_10_* = 26.98), and SP2 (*M* = 0.66, *SE* = 0.03) and SP3 (*p* = .033, *BF_10_* = 5.68). The difference between SP1 and SP2 approached significance (*p* = .057, *BF_10_* = 2.84).

1. **Experiment 2 further analysis: questionnaire**

| Mean perceived effect (and SE) of prioritization at SP1 (where the manipulation was targeted) and at other SPs at WM and LTM. Possible values are between -4 and +4, where 0 = no effect, positive values reflect a perceived positive effect, and negative values reflect a perceived negative effect. | | |
| --- | --- | --- |
|  | Effect at SP1 (where manipulation was targeted) | Effect at other SPs |
| WM | 1.42 (0.32) | -1.17 (0.37) |
| LTM | 1.13 (0.39) | -1.25 (0.35) |

**7. Cross-experimental analyses**

**Working memory**

*Accuracy*

Mean proportion correct (and SE) is displayed in Figure S.7.1A as a function of probe value and SP. A 2 (Probe value: Differential vs Equal) x 4 (SP: 1-4) within-subjects ANOVA revealed a marginally significant effect of probe value (*F*(1, 57) = 4.36, *MSE* = 0.01, *p* = .041*,* $\eta_{p}^{2}$ = .07), whereby participants performed more accurately in the equal value condition (*M* = .81, *SE* = .01) than the differential value condition (*M* = .79, *SE* = .01). However, the BF was slightly in favour of the null (*BF_10_* = 0.61, *BF_01_* = 1.63). A significant effect of SP emerged (*F*(3, 171) = 15.81, *MSE* = 0.02, *p* < .001*,* $\eta_{p}^{2}$ = .22; *BF_10_* > 10,000). Pairwise comparisons (corrected using Bonferroni-Holm) revealed that performance at SP1 (*M* = .84, *SE* = .01) was significantly better than performance at SP2 (*M* = .74, *SE* = .02; *p* < .001; *BF_10_* = 3934.50) and SP3 (*M* = .78, *SE* = .01; *p* = .003; *BF_10_* = 10.17). Performance at SP4 (*M* = .85, *SE* = .02) was also significantly better than performance at SP2 (*p* < .001; *BF_10_* > 10,000) and SP3 (*p* < .001; *BF_10_* = 1273.88). There was a significant interaction between probe value and SP (*F*(3, 171) = 13.49, *MSE* = 0.02, *p* < .001*,* $\eta_{p}^{2}$ = .19; *BF_10_* > 10,000). The BF analysis revealed the best model contained a main effect of SP and an interaction between probe value and SP (*BF_10_* > 10,000 relative to the null model containing participant only).


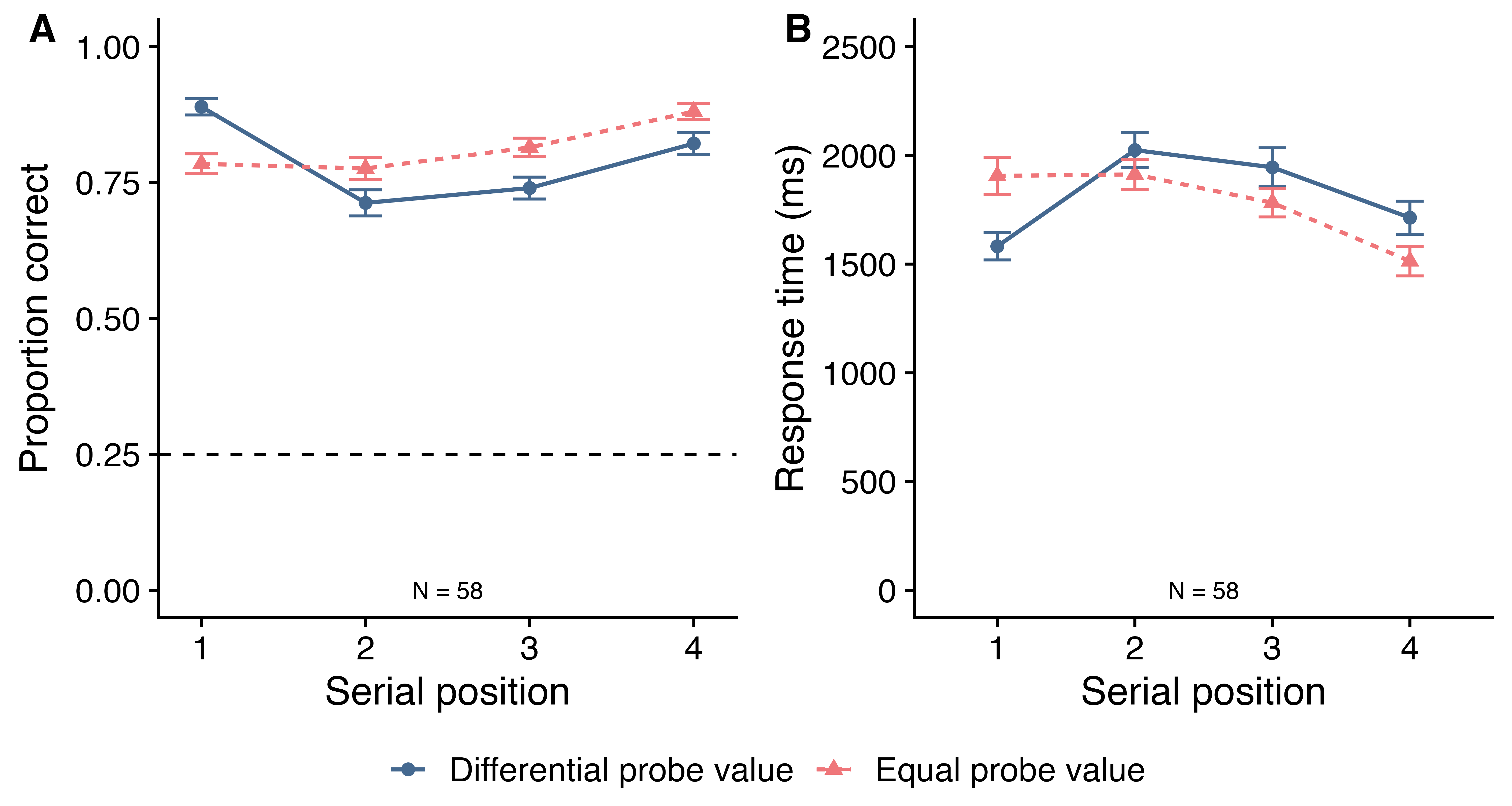


Figure S.7.1. Mean accuracy (and SE; Panel A) and RT (and SE; Panel B) across all participants in both experiments, as a function of probe value and SP.

To understand the interaction, Bonferroni-Holm corrected post-hoc tests were conducted to examine the effect of probe value at each SP (see Table S.7.1 for the means, SE, and t-test outcomes (including Bonferroni-Holm corrected p-values). To summarise, accuracy was significantly higher in the differential probe value condition relative to the equal probe value condition at SP1. The reverse pattern was observed at SP2, SP3 and SP4.

| *Table S.7.1. Mean accuracy, SE and t-test outcomes for the comparison between differential and equal probe value conditions in the WM phase.* | | | | | | | |
| --- | --- | --- | --- | --- | --- | --- | --- |
| SP | Differential mean (SE) | Equal mean (SE) | *t* | *df* | *p* | *d* | *BF10* |
| SP1 | 0.89 (0.02) | 0.78 (0.02) | 4.84 | 57 | < .001 | 0.64 | 1827.71 |
| SP2 | 0.71 (0.02) | 0.78 (0.02) | -2.33 | 57 | .023 | -0.31 | 1.72 |
| SP3 | 0.74 (0.02) | 0.82 (0.02) | -3.12 | 57 | .006 | -0.41 | 10.63 |
| SP4 | 0.82 (0.02) | 0.88 (0.01) | -3.23 | 57 | .006 | -0.42 | 14.35 |

*Response times*

Mean RT (and SE) is displayed in Figure S.7.1B as a function of probe value and SP. A 2 (Probe value: differential vs equal) x 4 (SP: 1-4) within-subjects ANOVA revealed no significant main effect of probe value (*F*(1, 57) = 0.82, *MSE* = 201676.74, *p* = .370*,* $\eta_{p}^{2}$ = .01; *BF_10_* = 0.19; *BF_01_* = 5.32). There was a significant main effect of SP (GG-corrected *F*(2.38, 135.55) = 18.07, *MSE* = 189811.64, *p* < .001*,* $\eta_{p}^{2}$ = .24; *BF_10_* > 10,000). Bonferroni-Holm corrected pairwise comparisons revealed that RT at SP1 (*M* = 1744, *SE* = 64.9) was significantly faster than RTs at SP2 (*M* = 1968, *SE* = 69.7; *p* < .001; *BF_10_* = 7182.05), SP3 (*M* = 1864, *SE* = 70.3; *p* = .047; *BF_10_* = 1.21), and SP4 (*M* = 1614, *SE* = 68.0; *p* = .047; *BF_10_* = 1.44). RT at SP4 was also significantly faster than at SP2 (*p* < .001; *BF_10_* > 10,000) and SP3 (*p* < .001; *BF_10_* > 10,000). Finally, RT at SP3 was significantly faster than at SP2 (*p* = .047; *BF_10_* = 3.70). There was also a significant interaction between probe value and SP (*F*(3, 171) = 19.31, *MSE* = 89297.21, *p* < .001*,* $\eta_{p}^{2}$ = .25; *BF_10_* > 10,000). The BF analysis that the best model included a main effect of SP and an interaction between probe value and SP (*BF_10_* > 10,000 relative to the null model containing participant only).

To understand the interaction, Bonferroni-Holm corrected post-hoc tests were conducted to examine the effect of probe value at each SP (see Table S.7.2 for the means, SE, and t-test outcomes). To summarise, RT was significantly faster in the differential probe value condition at SP1. The reverse was observed at SP3 and SP4. At SP2, there was no significant difference, but a trend towards faster in the equal probe value condition. BF analysis was slightly in favour of no effect at this SP.

| *Table S.7.2. Mean RT, SE and t-test outcomes for the comparison between differential and equal probe value conditions in the WM phase.* | | | | | | | |
| --- | --- | --- | --- | --- | --- | --- | --- |
| SP | Differential mean (SE) | Equal mean (SE) | *t* | *df* | *p* | *d* | *BF10* |
| SP1 | 1582 (62.8) | 1906 (85.9) | -4.24 | 57 | < .001 | -0.56 | 263.04 |
| SP2 | 2024 (80.8) | 1913 (69.9) | 1.92 | 57 | .060 | 0.25 | 0.80 |
| SP3 | 1945 (89.5) | 1782 (65.0) | 2.37 | 57 | .042 | 0.31 | 1.89 |
| SP4 | 1713 (76.0) | 1514 (67.8) | 4.22 | 57 | <.001 | 0.55 | 242.10 |

**Long-term memory**

*Accuracy*

Mean accuracy in the LTM phase is displayed in Figure S.7.2A as a function of probe value, SP, and tested-at-WM. A 2 (Probe value: differential vs equal) x 4 (SP: 1-4) x 2 (Tested-at-WM: not tested vs tested) within-subjects ANOVA revealed no significant effects of probe value (*F*(1, 57) = 0.15, *MSE* = 0.02, *p* = .699*,* $\eta_{p}^{2}$ < .01; *BF_10_* = 0.08; *BF_01_* = 12.44) or SP (*F*(3, 171) = 1.75, *MSE* = 0.02, *p* = .158*,* $\eta_{p}^{2}$ = .03; *BF_10_* = 0.05; *BF_01_* = 20.64). A significant main effect of tested-at-WM emerged (*F*(1, 57) = 331.92, *MSE* = 0.03, *p* < .001*,* $\eta_{p}^{2}$ = .85; *BF_10_* > 10,000), with higher accuracy for items that had been tested at WM (*M* = .62, *SE* = .02) relative to items that had not being tested (*M* = .41, *SE* = .01). There was a significant interaction between SP and tested-at-WM (*F*(3, 171) = 8.05, *MSE* = 0.02, *p* < .001*,* $\eta_{p}^{2}$ = .12; *BF_10_* = 239.00). No other interactions were significant (*F* ≤ 1.98, p ≥ .119, $\eta_{p}^{2}$ ≤ .03, *BF_10_* ≤ 0.19; *BF_01_ ≥* 5.31). The BF analysis indicated that the best model included a main effect of tested-at-WM, and a SP and tested-at-WM interaction (*BF_10_* > 10,000 relative to the null model containing participant only).


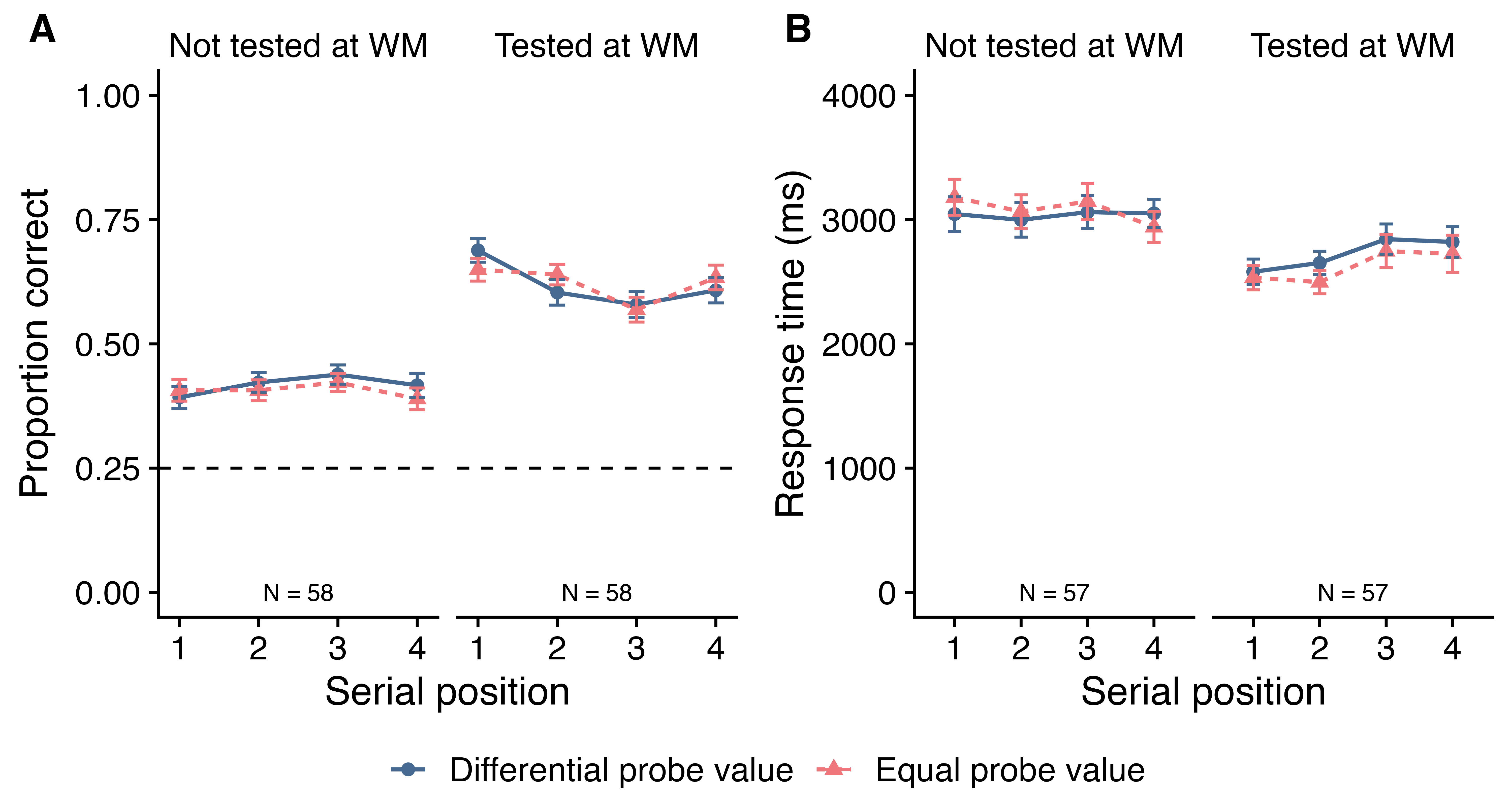


*Figure S.7.2.* Mean accuracy (Panel A) and RT (Panel B) in the LTM phase, as a function of probe value, SP, and tested-at-WM. Error bars represent SE.

To investigate this interaction between SP and tested-at-WM, one-way ANOVAs were conducted, followed by post-hoc tests as appropriate. When the item had not been tested at WM, there was no significant effect of SP (*F*(3, 171) = 1.07, *MSE* = 0.01, *p* = .365*,* $\eta_{p}^{2}$ = .02, *BF_10_* = 0.08, *BF_01_* = 12.59). When items that had been tested at WM, there was a significant effect of SP (*F*(3, 171) = 7.91, *MSE* = 0.01, *p* < .001*,* $\eta_{p}^{2}$ = .12, *BF_10_* = 333.63). Post-hoc tests revealed higher accuracy at SP1 (*M* = .67, *SE* = .02) than SP2 (*M* = .62, *SE* = .02; *p* = .027; *BF_10_* = 6.10) and SP3 (*M* = .57, *SE* = .02; *p* < .001; *BF_10_* = 5683.28). Accuracy at SP2 was marginally significantly lower than at SP3 (*p* = .048; *BF_10_* = 3.05). The difference between SP1 and SP4 (*M* = .62, *SE* = .02) approached significance (*p* = .070; *BF_10_* = 1.72), as did the between SP3 and SP4 both approached significance (*p* = .070; *BF_10_* = 1.63).

*Response times*

One participant was excluded from this analysis as they had an empty cell resulting from all data for that condition being excluded. This analysis was therefore conducted on data from 57 participants. RTs are displayed in Figure S.7.2B a function of probe value, SP, and tested-at-WM. A 2 (Probe value: differential vs equal) x 4 (SP: 1-4) x 2 (Tested-at-WM: yes vs no) within-subjects ANOVA revealed no significant main effect of probe value (*F*(1, 56) = 0.40, *MSE* = 427840.45, *p =* .532, $\eta_{p}^{2}$ = .01; *BF_10_* = 0.09; *BF_01_* = 10.56) or SP (*F*(3, 168) = 2.61, *MSE* = 355813.51, *p* = .053, $\eta_{p}^{2}$ = .05; *BF_10_* = 0.13, *BF_01_* = 7.67). There was a main effect of tested-at-WM (*F*(1, 56) = 86.50, *MSE* = 392384.91, *p* < .001, $\eta_{p}^{2}$ = .61; *BF_10_* > 10,000), with participants responding significantly faster when the item was tested at WM (*M* = 2675*, SE* = 93.6) relative to when it was not tested at WM (*M* = 3060, *SE* = 110.7). A significant interaction emerged between probe value and tested-at-WM (*F*(1, 56) = 4.89, *MSE* = 237488.21, *p* = .031, $\eta_{p}^{2}$ = .08), although the BF analysis was slightly in favour of no effect *(BF_10_* = 0.53, *BF_01_* = 1.89). A significant interaction emerged between SP and tested-at-WM *F*(3, 168) = 3.45, *MSE* = 366243.17, *p* = .018, $\eta_{p}^{2}$ = .06; *BF_10_* = 1.25). There were no other significant interactions (*F* ≤ 0.63, p ≥ .594, $\eta_{p}^{2}$ ≤ .01, *BF_10_* ≤ 0.04; *BF_01_ ≥* 26.86). The BF analysis revealed that the best model included tested-at-WM, and the tested-at-WM and SP interaction (*BF_10_* > 10,000 relative to the null model containing participant only).

To investigate the interaction between probe value and tested-at-WM, t-tests were conducted to investigate whether an effect of probe value emerged in the tested and not tested conditions, averaging over SP. No probe value effect emerged, both when the items were tested-at-WM (*t*(56) = 2.09, *p* = .082, *d* = 0.28, *BF_10_* = 1.09) and when they were not tested at WM (*t*(56) = -0.73, *p* = .467, *d* = -0.10, *BF_10_* = 0.19, *BF_01_* = 5.35).

To investigate this interaction between SP and tested-at-WM, one-way ANOVAs were conducted, followed by post-hoc tests as appropriate. When the item had not been tested at WM, there was no significant effect of SP (*F*(3, 168) = 0.99, *MSE* = 183961.89, *p* = .399*,* $\eta_{p}^{2}$ = .02, *BF_10_* = 0.07, *BF_01_* = 13.72). In contrast, when items that had been tested at WM, there was a significant effect of SP (GG-corrected *F*(2.51, 140.48) = 5.16, *MSE* = 211760.42, *p* = .004*,* $\eta_{p}^{2}$ = .08, *BF_10_* = 12.19). Post-hoc tests revealed significant differences between SP1 (*M* = 2556; *SE* = 89.0) and SP3 (*M* = 2795, *SE* = 116.3; *p* = .012; *BF_10_* = 14.88), and SP2 (*M* = 2574, *SE* = 83.6) and SP3 (*p* = .024; *BF_10_* = 6.70). The differences between SP1 and SP4 (*M* = 2773, *SE* = 126.0; *p* = .053; *BF_10_* = 2.81), and SP2 and SP4 (*p* = .085; *BF_10_* = 1.47) approached significance.
